# *Hydra* Mesoglea Proteome Identifies Thrombospondin as a Conserved Component Active in Head Organizer Restriction

**DOI:** 10.1038/s41598-018-30035-2

**Published:** 2018-08-06

**Authors:** Mark Lommel, Jennifer Strompen, Andrew L. Hellewell, Gnana Prakash Balasubramanian, Elena D. Christofidou, Andrew R. Thomson, Aimee L. Boyle, Derek N. Woolfson, Kane Puglisi, Markus Hartl, Thomas W. Holstein, Josephine C. Adams, Suat Özbek

**Affiliations:** 1 University of Heidelberg, Centre for Organismal Studies, Department of Molecular Evolution and Genomics, Im Neuenheimer Feld 230, 69120 Heidelberg, Germany; 2 School of Biochemistry, University of Bristol, Biomedical Sciences Building, University Walk, Bristol, BS8 1TD UK; 3 School of Chemistry, Cantock’s Close, University of Bristol, Bristol, BS8 1TS UK; 4 Institute of Biochemistry and Center for Molecular Biosciences, University of Innsbruck, Innrain 80-82, A-6020 Innsbruck, Austria; 5 Present Address: G200 Division of Applied Bioinformatics, German Cancer Research Institute (DKFZ) and National Center for Tumor Diseases (NCT) Heidelberg, Im Neuenheimer Feld 580, D-69120 Heidelberg, Germany; 6 Present Address: School of Chemistry, University of Glasgow, Joseph Black Building, University Avenue, Glasgow, G12 8QQ Scotland; 7 Present Address: Leiden Institute of Chemistry, Leiden University, POB 9502, NL-2300 RA Leiden, Netherlands

## Abstract

Thrombospondins (TSPs) are multidomain glycoproteins with complex matricellular functions in tissue homeostasis and remodeling. We describe a novel role of TSP as a Wnt signaling target in the basal eumetazoan *Hydra*. Proteome analysis identified *Hydra magnipapillata* TSP (HmTSP) as a major component of the cnidarian mesoglea. In general, the domain organization of cnidarian TSPs is related to the pentameric TSPs of bilaterians, and in phylogenetic analyses cnidarian TSPs formed a separate clade of high sequence diversity. *HmTSP* expression in polyps was restricted to the hypostomal tip and tentacle bases that harbor Wnt-regulated organizer tissues. In the hypostome, *HmTSP-* and *Wnt3*-expressing cells were identical or in close vicinity to each other, and regions of ectopic tentacle formation induced by pharmacological β-Catenin activation (Alsterpaullone) corresponded to foci of *HmTSP* expression. Chromatin immunoprecipitation (ChIP) confirmed binding of *Hydra* TCF to conserved elements in the *HmTSP* promotor region. Accordingly, β-Catenin knockdown by siRNAs reduced normal *HmTSP* expression at the head organizer. In contrast, knockdown of *HmTSP* expression led to increased numbers of ectopic organizers in Alsterpaullone-treated animals, indicating a negative regulatory function. Our data suggest an unexpected role for HmTSP as a feedback inhibitor of Wnt signaling during *Hydra* body axis patterning and maintenance.

## Introduction

Cnidarians are simple invertebrate metazoans that live in aquatic environments and are estimated to have diverged from the ancestor of bilaterian animals around 750 MYA^1^. *Hydra* species have been studied experimentally for many years for their simplicity of tissue organization and remarkable regeneration properties; such studies have provided insights into fundamental, conserved molecular processes that underlie body plan organization, tissue differentiation, regeneration and stem cell properties^2^. The attractiveness of *Hydra* as a model organism is further enhanced by the appreciation that cnidarians have a profile of protein-coding genes closer to that of vertebrates than do *D. melanogaster* or *C. elegans*^3–5^.

In *Hydra*, as in all cnidarians, the body wall consists of two cell layers, the ectoderm and endoderm, separated by an ECM layer, the mesoglea. Ultrastructurally, the mesoglea includes a basement membrane-like structure underneath each cell layer and a central, fibril-rich region that is morphologically reminiscent of the connective tissue ECM of vertebrates^6^. Several fibrillar collagens, collagen IV and laminin subunits have been identified by biochemistry and molecular cloning methods as components of the mesoglea^7–10^. Thus, *Hydra* mesoglea is a relevant and important model to understand fundamental properties and roles of ECM that may be conserved between basal metazoans and mammals.

Analysis *in silico* of the *H. magnipapillata* genome-predicted proteome for homologues of the major ECM proteins and adhesion receptors of vertebrates raised the possibility that the mesoglea might include additional conserved ECM components^11,12^. Importantly, the mesoglea of *Hydra* is acellular and can readily be separated from the adjoining cell layers as a sheet-like structure^13,14^. By undertaking the first proteomic study of isolated *Hydra* ECM, we identified *Hydra* thrombospondin as a prominent novel component of the mesoglea. In mammals, thrombospondins are matri-cellular, calcium-binding extracellular glycoproteins that have multi-faceted roles in cell-ECM interactions and cell signaling and are present both in basement membranes and connective tissue ECM^15^. Here, we show that *Hydra* TSP shares domains and oligomerization properties with bilaterian pentameric TSPs and exerts an unexpected role in *Hydra* body patterning as a negative feedback regulator of Wnt/β-Catenin-dependent organizer formation.

## Results

### *Hydra* TSP is a major component of the mesoglea

**Figure 1:**
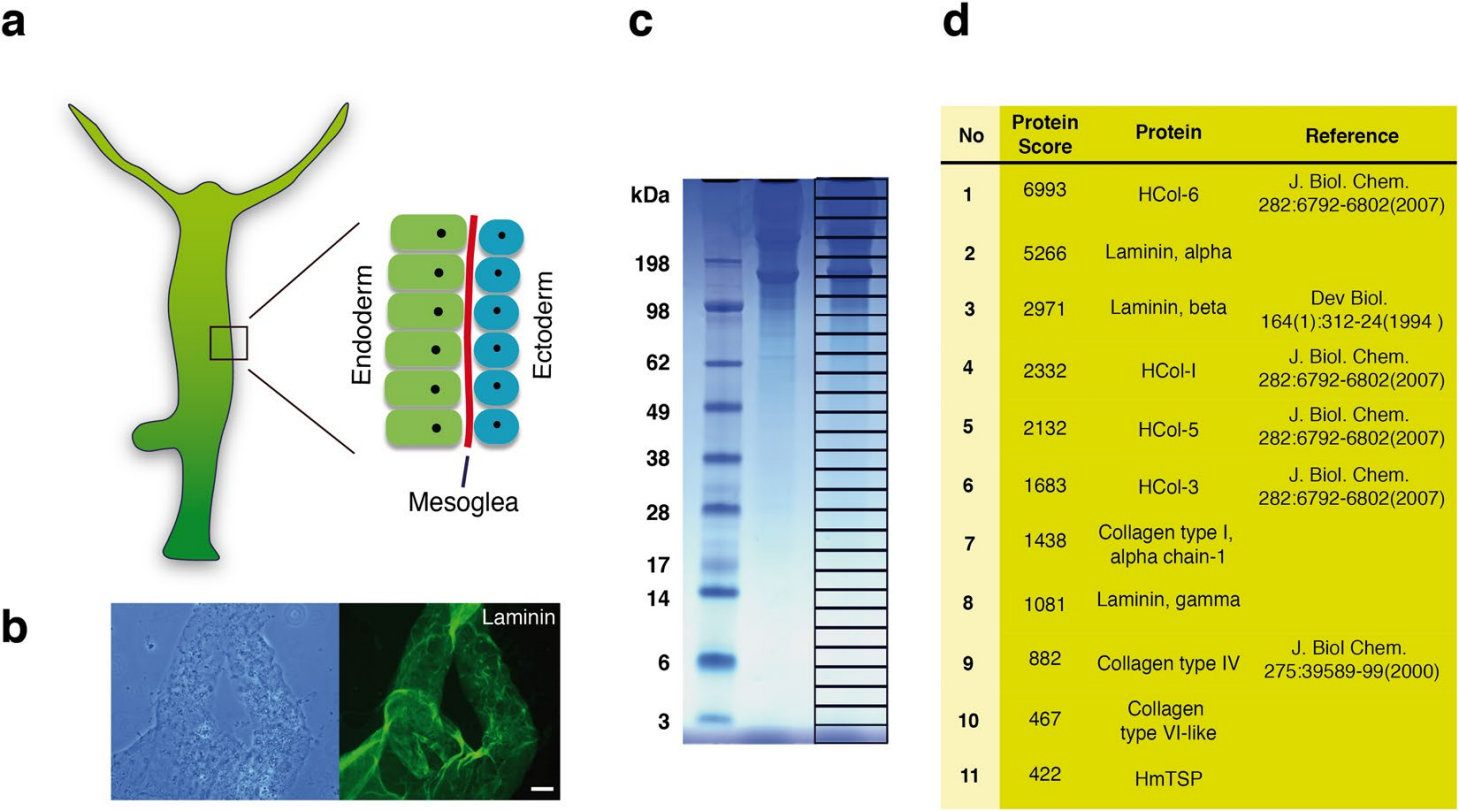
*Hydra* mesoglea extraction and proteome analysis. (**a**) Schematic representation of *Hydra* mesoglea. The body wall of *Hydra* is a bilayer of endo- and ectodermal tissues connected by a thin extracellular matrix, the mesoglea. (**b**) Immunostaining of isolated mesoglea with monoclonal antibody to *Hydra* laminin. Left panel shows a phase contrast image. Bar is 500 μm. (**c**) One-dimensional SDS-PAGE analysis of isolated *Hydra* mesoglea. The 27 gel slices used for mass spectrometry are indicated. (**d**) The highest-scoring mesoglea proteins identified in the analysis include HmTSP. References are given for proteins previously characterized at the protein or mRNA level. The full dataset is given in Supplementary Table S1.

The cnidarian mesoglea is an ECM that supports the epithelial bilayer of the body tissue^6^ (Fig. 1a). In *Hydra*, it can be isolated as an acellular sheet that is rich in collagens and laminin (shown for laminin in Fig. 1b). To define globally the major protein constituents of *Hydra* ECM, we performed an unbiased proteomic analysis of isolated mesoglea. A total of 80 μg of mesoglea extract was solubilized in SDS-PAGE sample buffer containing dithiothreitol and separated by one-dimensional SDS-PAGE (Fig. 1c). Gel slices (n = 27) were subjected to Orbitrap mass spectrometry analysis following trypsin/collagenase treatment. Annotation of the obtained mesoglea peptide sequences from genomic and transcriptomic databases and the application of a MASCOT protein score threshold of 40 yielded 37 unique proteins, including core matrisome proteins that were described previously by expression analysis (Fig. 1d and full list in Supplementary Table S1). *Hydra magnipapillata* thrombospondin (HmTSP) was identified as a prominent novel component (Fig. 1d). The identification of HmTSP was substantiated by BLAST analyses of the *H. magnipapillata* genome and transcriptome. HmTSP is predicted to be an 824aa polypeptide with extensive sequence homology to mammalian thrombospondins (e-value 2e-169 and 54% identity to human TSP5, 53% identity to human TSP3). In BLASTP searches of GenBank proteins, HmTSP is most closely related to the TSPs of insects, e.g. *Anopheles gambiae* TSP (66% coverage, e-value 0.0, 54% sequence identity).

### Cnidarian TSPs exhibit considerable sequence diversity

**Figure 2:**
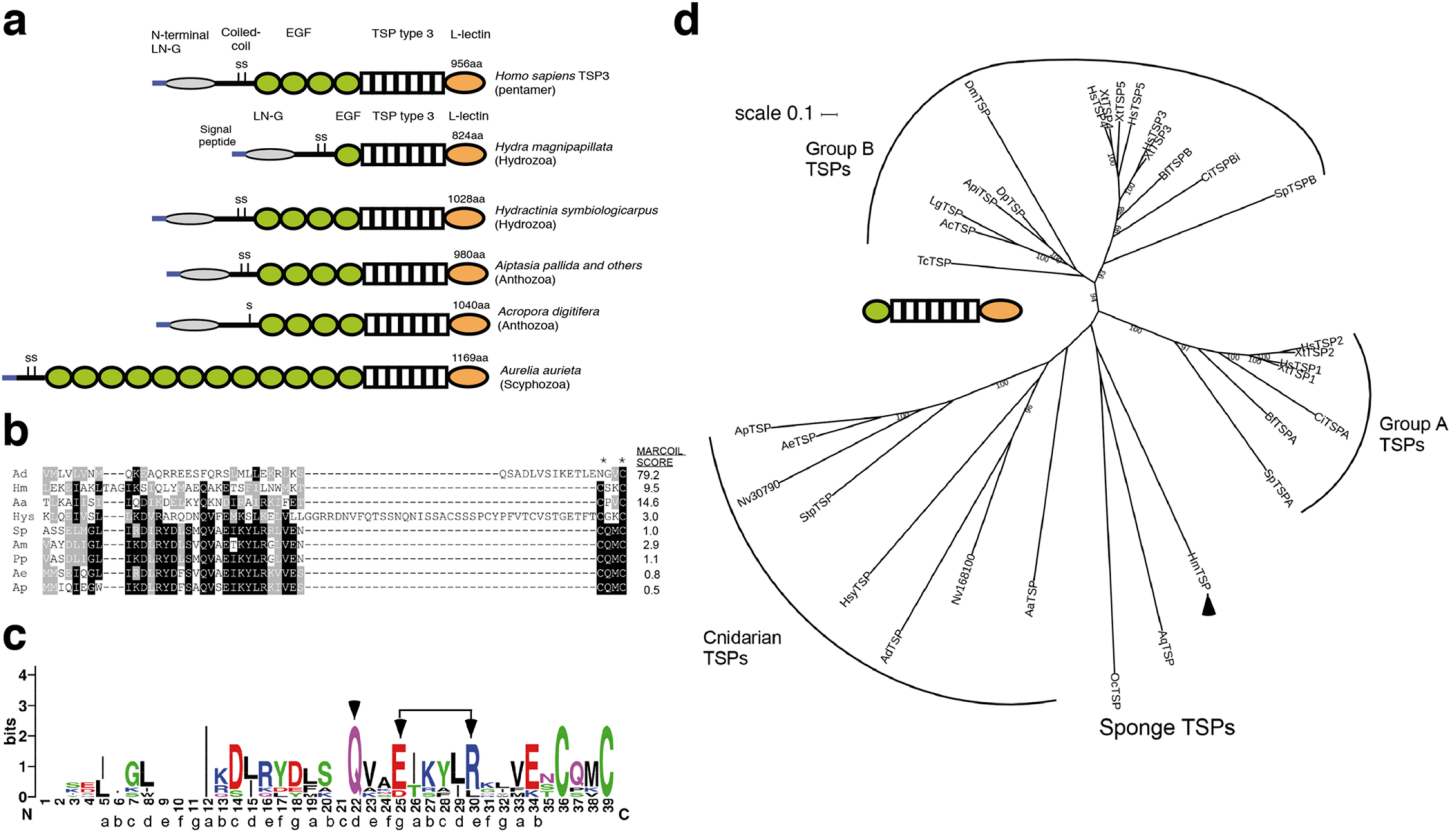
Domain structures and phylogeny of cnidarian TSPs. (**a**) Schematic diagrams of the domain architecture of human TSP3 and TSPs identified from cnidarians representative of several classes. (**b**) Multiple sequence alignment of the putative oligomerization regions from cnidarian TSPs. The alignment was prepared in MUSCLE 3.8 and is presented in Boxshade. Black shading indicates residues conserved in over 50% of the sequences; grey shading indicates semi-conservative substitutions. Asterisks indicate the conserved Cys residues. MARCOIL scores are the mean coiled-coil probability score/residue calculated from the sum of all score/residue divided by the number of residues in the sequence. Key: Aa = *Aurelia aurita*, Ad = *Acropora digitifera*, Ae = *Anthopleura elegantissima*, Am = *Acropora millepora*, Ap = *Aiptasia pallida*, Hm = *Hydra magnipapillata*, Hys = *Hydractinia symbiolongicarpus*, Pa = *Porites australiensis*, Sp = *Stylophora pistillata*. (**c**) Sequence Logo based on the multiple sequence alignment shown in B, after removal of the region unique to *H. symbiolongicarpus*. At each position, the bit score for each residue indicates the strength of conservation. Arrows indicate the well-conserved central Q and EXXXXR motif, which are also conserved features of the coiled-coil domains of pentamerizing TSPs from bilaterians^16^. A notional heptad repeat phase, based on these features, is shown underneath the Logo. (**d**) Phylogenetic tree of TSPs. The unrooted maximum-likelihood tree was prepared in PhyML from a MUSCLE multiple sequence alignment (552 positions) of the indicated C-terminal region of 33 TSPs from cnidarians, representative bilaterians and two sponges. *Hydra* TSP is indicated by the arrowhead. Nodes with bootstrap support >0.85 are indicated. Scale bar indicates substitutions/site.

The predicted HmTSP protein is most similar in domain organization to the subgroup B pentameric TSP of bilaterians and includes a signal peptide, laminin G domain-like N-terminal domain, thrombospondin type 3 repeats, and a C-terminal L-lectin-like domain (Fig. 2a). However, HmTSP is unusual in having only a single EGF-like domain and in lacking a distinct coiled-coil region between the N-terminal domain and EGF domain; in most TSPs a coiled-coil sequence at this position mediates oligomerization^16^ (shown for TSP3 in Fig. 2a). To better understand these distinctions, we examined cnidarian thrombospondins more widely. Many cnidarians encode a single TSP, each with the typical domain organization of a subgroup B TSP that includes four tandem EGF-like domains; however, a unique form of TSP was identified in the scyphozoan *Aurelia aurita* (moon jellyfish), which lacks the N-terminal domain and includes twelve repeated EGF-like domains (Fig. 2a). Unlike the cnidarian species examined here, *Nematostella vectensis* is unusual in encoding four TSP-like sequences, all of which are transcribed^17^. We found that, with the exception of *Acropora digitifera* TSP, cnidarian TSPs are diverse in the expected ‘oligomerization region”, with sequences of varying length that are not predicted to form coiled-coils (Fig. 2b, only *A. digitifera* TSP has a MARCOIL score predictive of a coiled-coil). In most cases, the region ends with a conserved Cys-X-X-Cys motif, equivalent to the motif present in subgroup B TSPs^15^ (Fig. 2b). A consensus Sequence Logo of the aligned sequences indicated that a centrally positioned glutamine (Q) and EXXXXR motif (both arrowed in Fig. 2c) are well-conserved. These are features that appear to be important for the pentamerization of TSP coiled-coil domains^16,18^. By applying a notional heptad repeat phasing^19^ to the cnidarian Sequence Logo, weak conservation of hydrophobic residues was apparent at the *a* and *d* positions of the heptad (Fig. 2c). Thus, even in the absence of clear coiled-coil character, this region of cnidarian TSPs includes several sequence features reminiscent of the coiled-coil domains of bilaterian TSPs.

The relationship of cnidarian TSPs to the wider TSP family was examined by phylogenetic analysis based on the conserved C-terminal region (i.e. from the last EGF domain to *C* terminus). The cnidarian and sponge TSPs formed a distinct sequence group, with in-clade sequence diversity (indicated by branch lengths) much greater than that within the TSP A (trimeric) or TSP B (pentameric) clades of TSPs from bilaterians (Fig. 2d). Notably, *Hydra* TSP (arrowed) is distinct from all the other cnidarian TSPs examined, such that it grouped, albeit with weak bootstrap support and possibly due to long-branch attraction, with sponge TSPs.

### *Hydra* Thrombospondin oligomerizes as a pentamer

**Figure 3:**
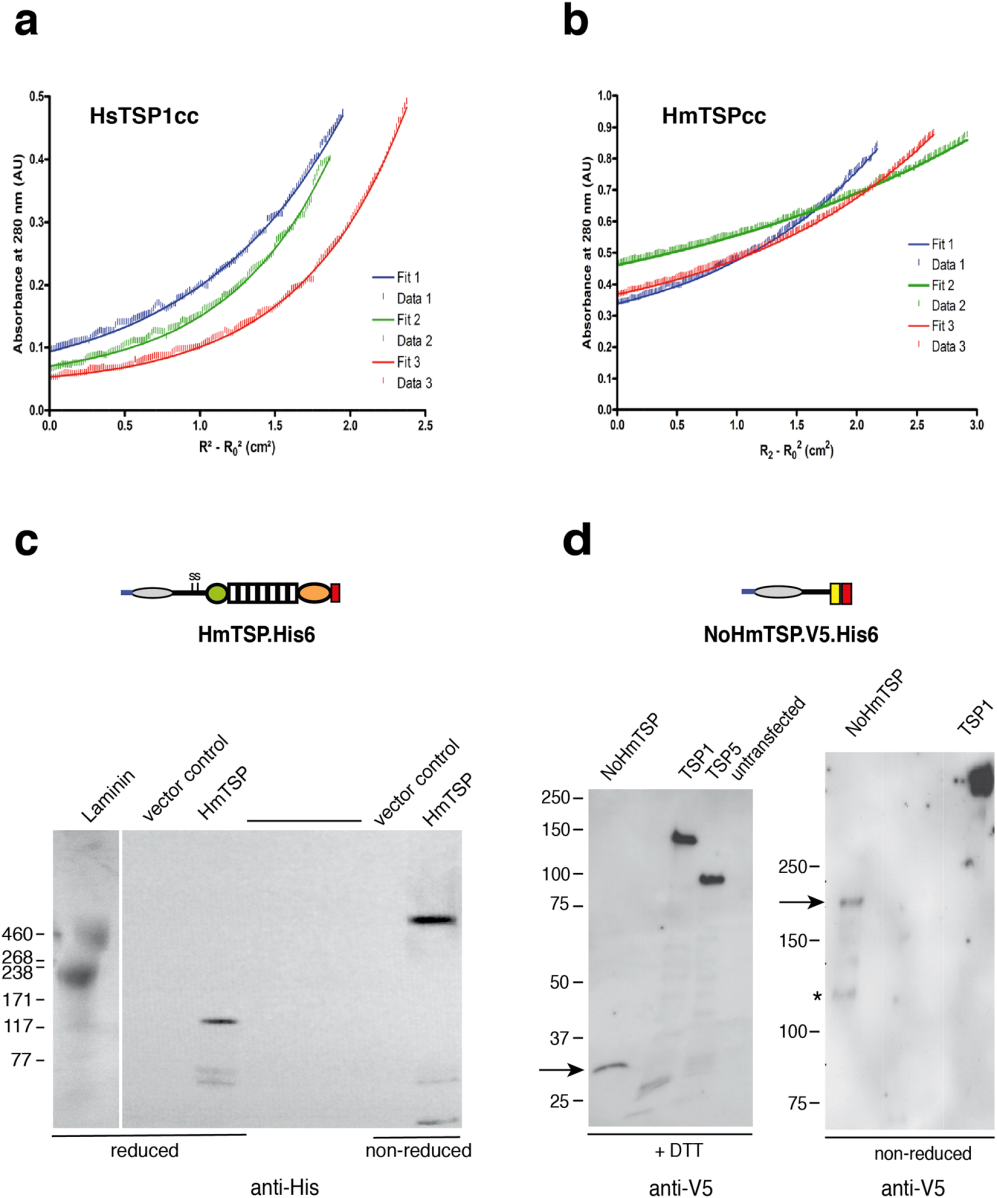
Oligomerization properties of HmTSP. (**a**,**b**) Analytical ultracentrifugation properties of HsTSP1cc (A) and HmTSPcc (B) peptides. Representative sedimentation equilibrium curves are shown at three different centrifugation speeds (data 1–3 in each graph). Peptides were dissolved in PBS at 50 µM (HsTSP1cc) or 400 µM (HmTSPcc) and analysis was carried out at 20 °C. Each dataset fitted well to a single, ideal species model (fit 1–3 in each graph) and returned molecular weights of 14800 Da for the HsTSP1cc (2.96 x monomer mass) and 9365 Da for HmTSPcc (2.98 x monomer mass). (**c**,**d**) Analysis of oligomerization of HmTSP. Schematic diagrams of the HmTSP proteins used in these experiments are given above the respective blot panels. In each panel, molecular mass markers are indicated in kDa. (**c**) Analysis of full-length HmTSP-His expressed in HEK293T cells by immunoblotting. Samples under nonreducing or reducing conditions as indicated were resolved on 4–20% polyacrylamide gels. 2 µg Laminin was loaded on the same gel as an additional molecular weight reference (Laminin A chain 400 kDa, Laminin B chains 200 and 210 kDa) and visualized by colloidal Comassie Blue staining. (**d**) Analysis of oligomerization for the *N*-terminal truncated protein NoHmTSP.V5 expressed in COS7 cells. To obtain the most accurate apparent molecular weight for non-reduced NoHmTSP.V5, reduced and non-reduced samples were run on separate gels and electrophoresis of the non-reduced samples was continued until certain molecular weight markers had been run off the gel. Arrows indicate the migration position in each analysis; asterisk in right panel indicates the second band consistent with a tetrameric form. The uncropped blot images for Fig. 3d are included in Supplementary Fig. S2a,b.

As noted, the TSPs of vertebrates assemble as trimers or pentamers and oligomerization is important for several functional activities^15^. To investigate the oligomerization properties of *Hydra* TSP, we first analyzed a 25aa synthetic peptide corresponding to HmTSP residues 249–273 (designated HmTSPcc) for oligomerization activity in comparison to the trimerizing coiled-coil domain of human TSP1 (designated HsTSP1cc; residues 275–314 of TSP1) (Supplementary Fig. S1a–c; quality control for the purified peptides is presented in Supplementary Fig. S1f-I). Circular dichroism (CD) spectroscopy of HsTSP1cc at two different concentrations was consistent with a predominantly α-helical structure and an increased CD signal at the higher concentration was consistent with oligomerization (Supplementary Fig. S1d). At 100 μM, the midpoint (T_M_) of the thermal unfolding transition detected by CD spectroscopy of HsTSP1cc was approximately 57 °C. CD spectra of the HmTSPcc peptide indicated poor folding at 10 μM and therefore data were obtained at 100 μM peptide only. At 5 °C, the CD spectrum of HmTSPcc was consistent with an α-helical structure. At 20 °C, (within the environmental temperature range for living *Hydra)*, the minima at 208 nm and 222 nm were less pronounced, indicating low thermal stability (Supplementary Fig. S1e). Analysis of sedimentation-equilibrium data recorded by analytical ultracentrifugation (AUC) returned single trimeric species for both peptides (Fig. 3a,b). In both cases, the residual data (the difference between raw and fitted data) were distributed randomly, indicating that there was no systematic error in the datasets and, thus, confirming trimeric species rather than equilibria with other oligomer states. Together, these biophysical data show that these relatively short peptide sequences can direct protein oligomerization, albeit to a structure of low thermal stability in the case of HmTSPcc.

To investigate oligomerization of full-length HmTSP, the protein was expressed recombinantly in HEK293T cells. The calculated molecular weight of the recombinant HmTSP was 97.47 kDa and under reducing SDS-PAGE a single band of an apparent molecular weight of ~115 kDa was detected in lysates of transfected cells. Under non-reducing conditions the protein migrated slower than the laminin alpha chain (400 kDa) and 460 kDa prestained marker band, indicative of an oligomer state larger than a trimer or tetramer (Fig. 3c). To assess the oligomerization state more accurately, a truncated protein was engineered that comprised the *N*-terminal domain plus the putative oligomerization region (aa 1–296, designated NoHmTSP.V5). The recombinant NoHmTSP.V5 secreted by COS7 cells was compared with previously analyzed human TSP1.V5 and TSP.V5^20^. Under reducing conditions, NoHmTSP.V5 protein migrated in consistence with the expected mature monomer molecular weight of 36.6 kDa, and TSP1 and TSP5 also migrated at their expected monomer molecular weights, calculated as 132.3 kDa (TSP1.V5) or 85.6 kDa (TSP5.V5) (Fig. 3d, left panel, Supplementary Fig. S2). Under the experimental conditions of matched transfection, harvesting of conditioned media, and gel-loading, the V5 antibody signal for NoHmTSP.V5 was consistently less strong than for TSP1.V5 or TSP5.V5 (Fig. 3d, left panel). Under non-reducing conditions, NoHmTSP.V5 was again detected more weakly than TSP1.V5. The major NoHmTSP.V5 band migrated with a molecular weight of ~189 kDa, consistent with the predicted molecular weight of a pentamer, 183.3 kDa. In many experiments, a minor band of approximately 141 kDa, consistent with a tetramer, was also detected (Fig. 3d, right panel, Supplementary Fig. S2). On the same gel, TSP1.V5 migrated in consistence with the predicted molecular weight of a trimer of 397 kDa. We conclude that HmTSP assembles as a pentamer, yet the oligomerization region in isolation is stable only as a trimer, implying that the paired cysteine residues (Fig. 2c) are important to control pentamer assembly.

### HmTSP expression is highly localized in adult polyps and bud stages

**Figure 4:**
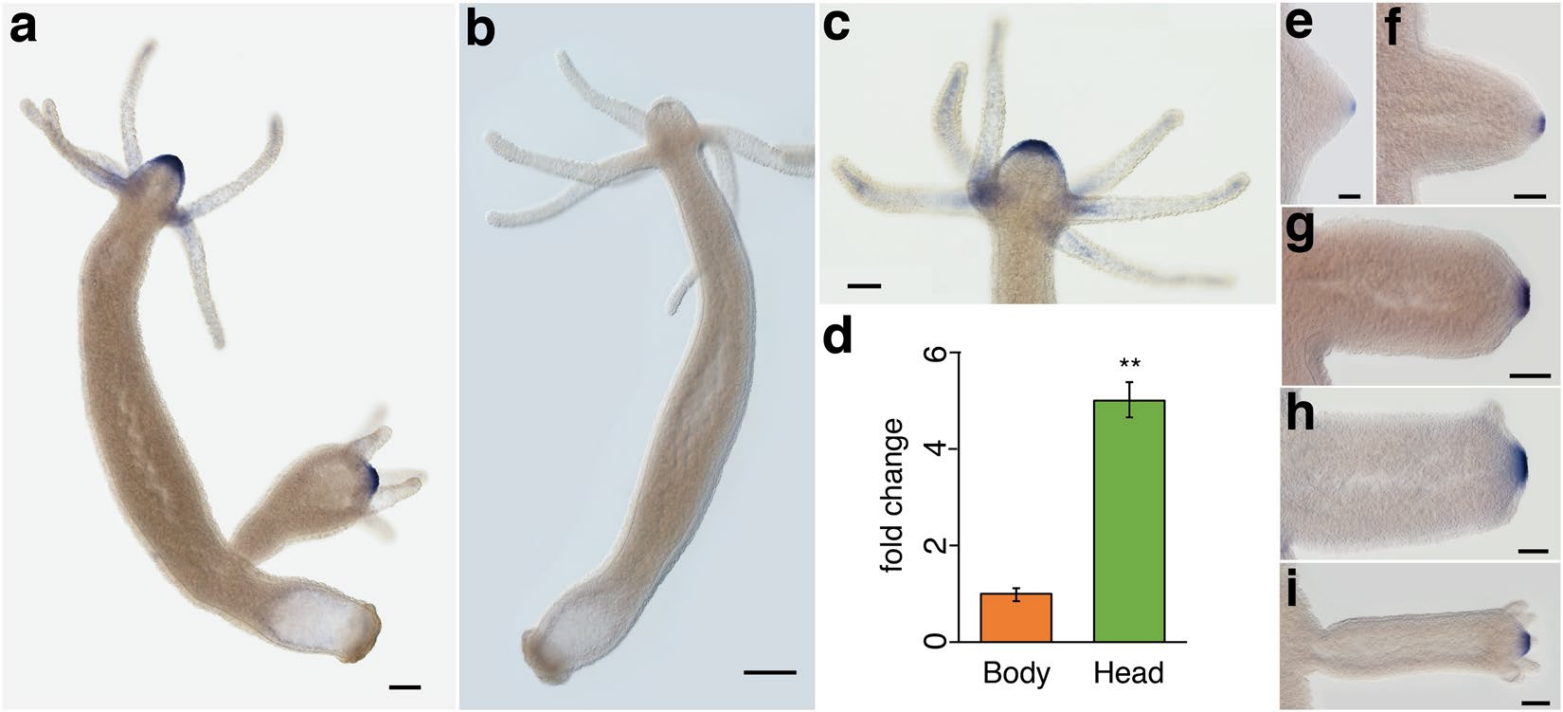
Expression pattern of *HmTSP* in adult *Hydra magnipapillata* polyps and budding stages. (**a**) *HmTSP* expression in adult polyps is restricted to the hypostome region and tentacle bases. (**b**) The sense control probe shows no specific hybridization. (**c**) *HmTSP* expression is ectodermal in the hypostome area and endodermal in cells of the tentacle bases. (**d**) Quantitative real-time PCR analysis of *HmTSP* expression in head and body tissues confirms the elevated expression in the head region. Results represent mean +/− S.D. from 3 independent experiments, analyzed by *t* tests. **p < 0.01. (**e**,**f**) *HmTSP* expression pattern in bud stages 3–7 according to Campbell^23^. Representative of 10 hydras examined. Scale bars: A: 400 µm, B: 500 µm, C:200 µm, E-I: 100 µm.

Major mesoglea components of *Hydra* including Hcol1, Hcol2, laminin subunits and Hcol4 are expressed along the length of the body column^8,9,21,22^. The mRNA expression pattern of *HmTSP* was examined by *in situ* hybridization (ISH) experiments on intact hydras. In adult polyps *HmTSP* had a strong spot-like expression in ectodermal cells of the hypostomal area (Fig. 4a,c and probe specificity control in Fig. 4b). Weaker expression was detected in endodermal epithelial cells of the tentacle with increasing intensity towards the tentacle bases (Fig. 4a,c). The restriction of *HmTSP* expression to the head region of *Hydra* was confirmed by quantitative RT-PCR on RNA from body and head tissues, which showed 4–5-fold higher levels in the head (Fig. 4d).

When *HmTSP* expression was monitored during budding, *Hydra’s* asexual reproduction process, no signal was detected until bud stage 3, which is characterized by a visible projection of both epithelial cell layers from the body of the parent (Fig. 4e)^23^. At this stage, *HmTSP* expression was first detected as a small spot at the ectodermal tip of the growing bud (Fig. 4e,f), which reached its full extension at bud stage 5, before the first tentacle rudiments become visible (Fig. 4g). Expression then remained constant throughout further development and up to the detachment of the new polyp (Fig. 4h,i). These gene expression data indicate a very localized pattern for *HmTSP* in a distinct spot of ectodermal epithelial cells around the mouth region of *Hydra*. This region is well-known as the hypostomal organizer that harbors the Wnt signaling center^24^.

### HmTSP expression parallels β-Catenin dynamics during head regeneration

**Figure 5:**
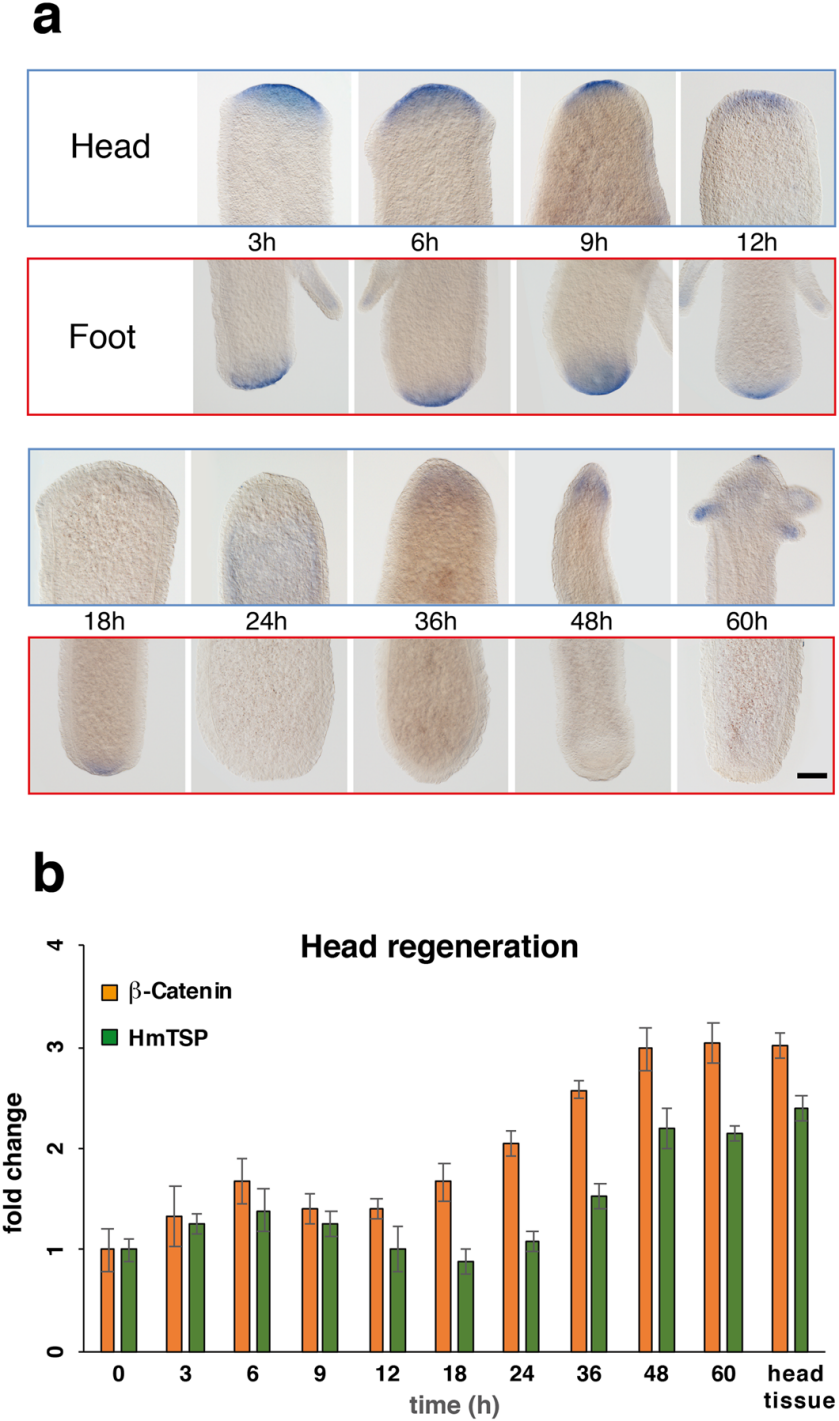
*HmTSP* expression during regeneration. (**a**) Animals were decapitated at 50% body length and *HmTSP* expression in the prospective head or foot regions was monitored via ISH on fixed specimens at the indicated time points (upper row, boxed in blue: head regenerates; lower row, boxed in red: foot regenerates). Representative of 10 hydras examined. Bar = 400 µm. (**b**) Quantitative real-time PCR analysis of *HmTSP* and *β-Catenin* expression during head regeneration. Results represent mean +/− S.D. from 3 independent experiments compared to time point 0.

To evaluate whether *HmTSP* could have a role in body axis formation we performed head and foot regeneration experiments. Animals were cut at 50% body length and *HmTSP* expression was monitored at different time points during regeneration of both fragments (Fig. 5a). Under these conditions, a new head is formed on the body column, or a new foot on the head fragment, within 36–48 hrs^25^. In both head and foot fragments, early up-regulation of *HmTSP* expression at the site of wound closure was apparent 3–9 hrs after transection (Fig. 5a). *HmTSP* expression was then lost from the regenerating head until the late patterning process of the new head (48 hrs), where it reappeared first at the tentacle buds. At later stages (60 hrs), the spot-like expression at the tip of the hypostome was recovered. In contrast, the foot region did not show *HmTSP* expression after 12 hrs. These data suggest a fast injury response followed by a slow recovery of the steady state *HmTSP* expression in the specific context of head re-patterning. Our findings are in line with a recent report demonstrating that a series of β-Catenin target genes are transiently up-regulated in foot regenerates of *Hydra*^26^. To confirm a patterning-dependent up-regulation of *HmTSP* transcripts we quantified the expression of *β-Catenin* and *HmTSP* during head regeneration (Fig. 5b). Both transcripts showed a slight up-regulation during the first injury response and a gradual up-regulation in the late phase of head regeneration reaching the steady-state expression level of head tissue at 48 h. Taken together, these data indicate a biphasic expression of *HmTSP* during head regeneration that is strongly reminiscent of *β-Catenin* expression dynamics.

### Regulation of HmTSP by β-Catenin signaling

**Figure 6:**
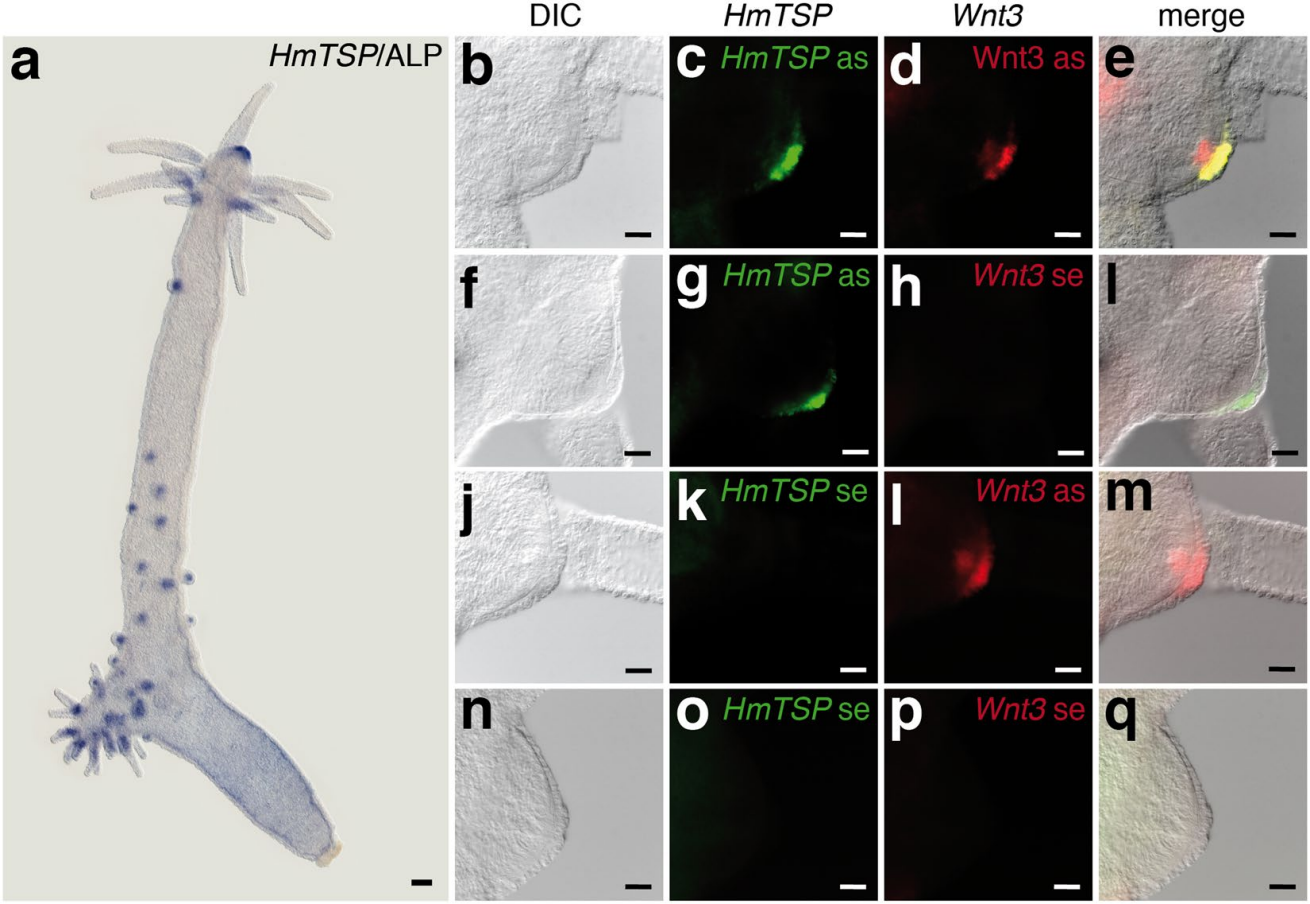
*HmTSP* expression correlates with *Wnt3*. (**a**) *HmTSP* expression is up-regulated at sites of ectopic tentacle formation, as determined by ISH 72 hrs after systemic treatment with 2.5 μM ALP. Representative of 10 hydras examined. Scale bar: 500 µm. (**b**–**q**) Dual color fluorescent ISH shows overlapping expression for *HmTSP* (green) and *Wnt3* (red) transcripts at the hypostome of steady state polyps (**b**–**e**). Combination of *HmTSP* antisense and *Wnt3* sense probes (**g**–**i**) and vice versa (**j**–**m**) as well as the use of both sense probes (**n**–**q**) confirmed the specific detection of *HmTSP* and *Wnt3* transcripts by the corresponding antisense probes. as, antisense probe; s, sense probe. Scale bars: 50 µm. Representative of 10 hydras examined.

Therefore, we next asked whether *HmTSP* is a target of β-Catenin in *Hydra*. Ectopic activation of the Wnt pathway can be induced by treating hydras with the glycogen synthase kinase-3β inhibitor ALP^27^. This leads to a global increase in nuclear β-Catenin that imparts head formation capacity on tissue of the gastric region, thereby inducing numerous ectopic tentacles. This process is accompanied by a spot-like expression of canonical Wnt ligands and Frizzled receptors^28^ as well as Wnt downstream targets such as *nodal-related*^29^. In animals treated with ALP, *HmTSP* was up-regulated in foci at ectopic tentacles, very similar to the pattern observed previously for *Wnt3*^30^ (Fig. 6a). Thus, *HmTSP* expression appears to depend on the status of Wnt pathway signalling activity. This notion was strongly supported by fluorescent dual colour ISH for *HmTSP* and *Wnt3*, that showed overlapping expression of the two transcripts in ectodermal cells of the hypostomal organizer (Fig. 6b–q). *Wnt3* was additionally expressed in a smaller patch of adjacent endodermal cells as also reported earlier for a transgenic Wnt3P::GFP line^30^ (Fig. 6l,m).

**Figure 7:**
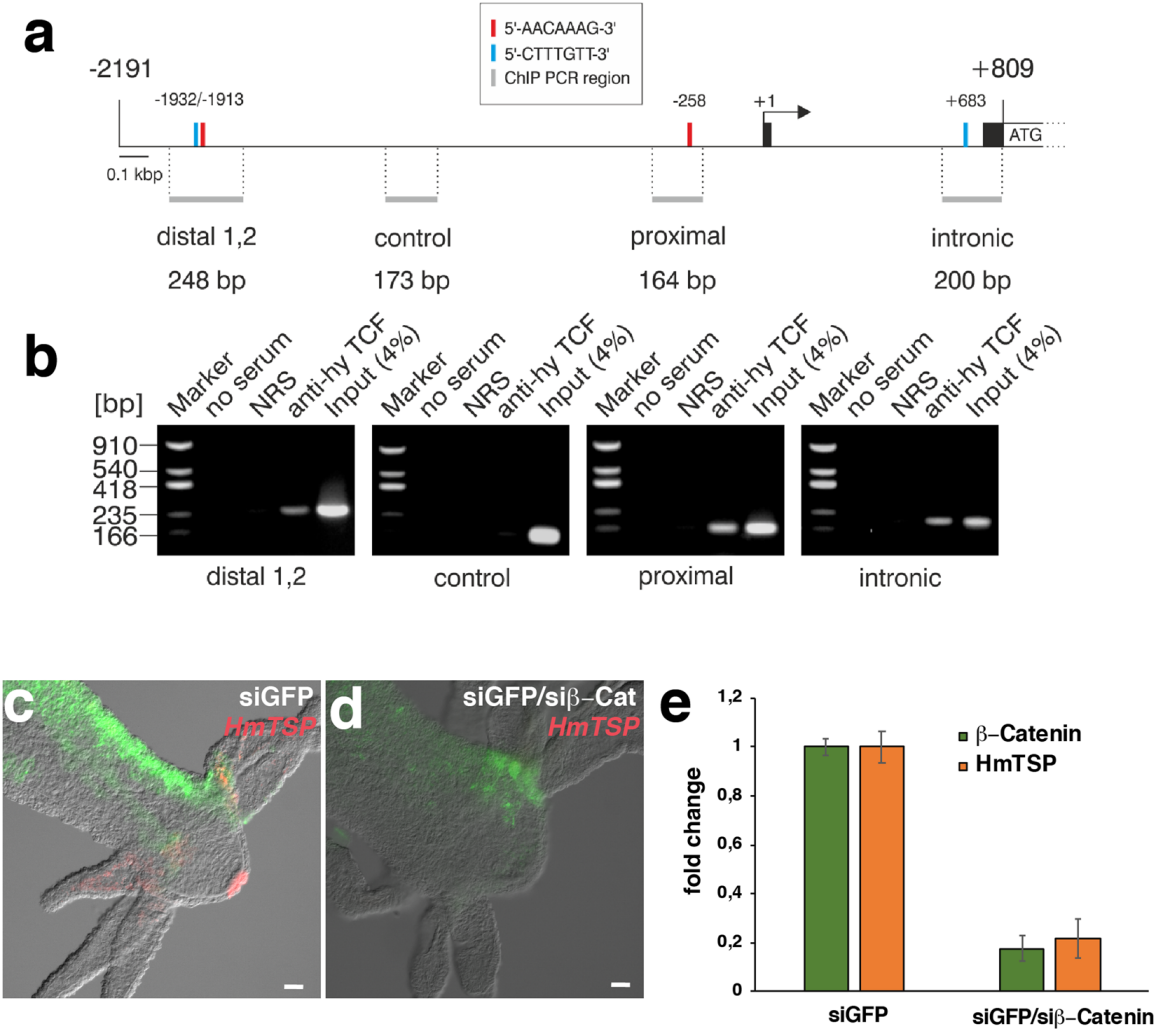
β-Catenin-dependent regulation of *HmTSP* expression. (**a**) Topography of the *HmTSP* promoter including -2191 bp of 5′ untranslated region. Black boxes depict the first two exons of the *HmTSP* gene. The arrow indicates the transcription start site of the *HmTSP* mRNA (accession no. XM_012702849), and ATG the translation start site. Positions of canonical TCF binding motifs are indicated by red (5′-CTTTGTT-3′) or blue (5′-AACAAAG-3′) bars, respectively. The localization and size of DNA segments flanked by specific ChIP primer pairs are indicated by horizontal grey bars. (**b**) ChIP analysis of the *HmTSP* promoter region using chromatin from whole *Hydra* animals. A polyclonal antibody directed against *Hydra* TCF was used for immunoprecipitation, followed by PCR amplification of the indicated fragments from the *HmTSP* regulatory region including a region without a TCF binding site (control). Reactions with normal rabbit serum (NRS) or total chromatin (Input) were used as further controls. PCR products were resolved by agarose gel electrophoresis and visualized by ethidium bromide staining. One representative of two replicates using independent chromatin preparations is shown (see Supplementary Fig. S3a). (**c**,**d**) ISH analysis of *HmTSP* expression (red) in the head regions of transgenic animals with ectodermal GFP expression (green) electroporated with control *GFP* siRNA (**c**) or *GFP* and *β-Catenin* siRNAs (**d**). *HmTSP* transcript was absent in electroporated areas with impaired *β-Catenin* expression. Note that GFP-positive areas indicate patches of tissue not affected by the electroporation. ISH was performed 8 days after electroporation with siRNAs. Scale bars = 50 µm. Representative of 10 hydras examined. **E**. Quantitative rt PCR analysis of *β-Catenin* and *HmTSP* expression upon siRNA mediated *β-Catenin* knockdown. Reduced expression of *β-Catenin* in siRNA-treated animals correlated with a major decrease in expression of *HmTSP*. Columns represent the mean and error bars the standard deviation from three independent experiments.

Upon examination of the promoter region of the *HmTSP* gene for regulatory elements, we identified four putative TCF binding elements (TBEs) with the conserved sequence motifs 5′CTTTGTT or 5′AACAAAG (Fig. 7a), similar to those previously characterized in the *Hydra Wnt3* promoter (CTTTGWW, W = A or T)^30^. This suggests that *HmTSP* can be part of the β-catenin/TCF-regulated feedback loop in Wnt3-expressing cells. To provide evidence for a direct regulation of *HmTSP* by β-catenin/TCF, we took advantage of a recently established chromatin immunoprecipitation (ChIP) assay using *Hydra* chromatin and a *Hydra* TCF-specific antiserum (anti-hyTCF)^26^. Chromatin immunoprecipitation followed by PCR with primers flanking each TBE in the *HmTSP* promotor resulted in specific PCR products encompassing all identified TBEs. No PCR product was obtained using primers comprising a control region without a TBE, suggesting that TCF is specifically bound to the TBE DNA regions in the *HmTSP* promoter (Fig. 7b, Supplementary Fig. S3a). Furthermore, down-regulation of *β-catenin* expression by electroporation of siRNA targeted to the *β-catenin* transcript (Fig. 7c–e), or pharmacological inhibition of β-catenin/TCF binding by iCRT14 (Supplementary Fig. S3b), led to a loss of *HmTSP* expression in the hypostome area. These data confirm that *HmTSP* is positively regulated by *β-catenin* activity *in vivo*.

### HmTSP acts as negative regulator of organizer formation

**Figure 8:**
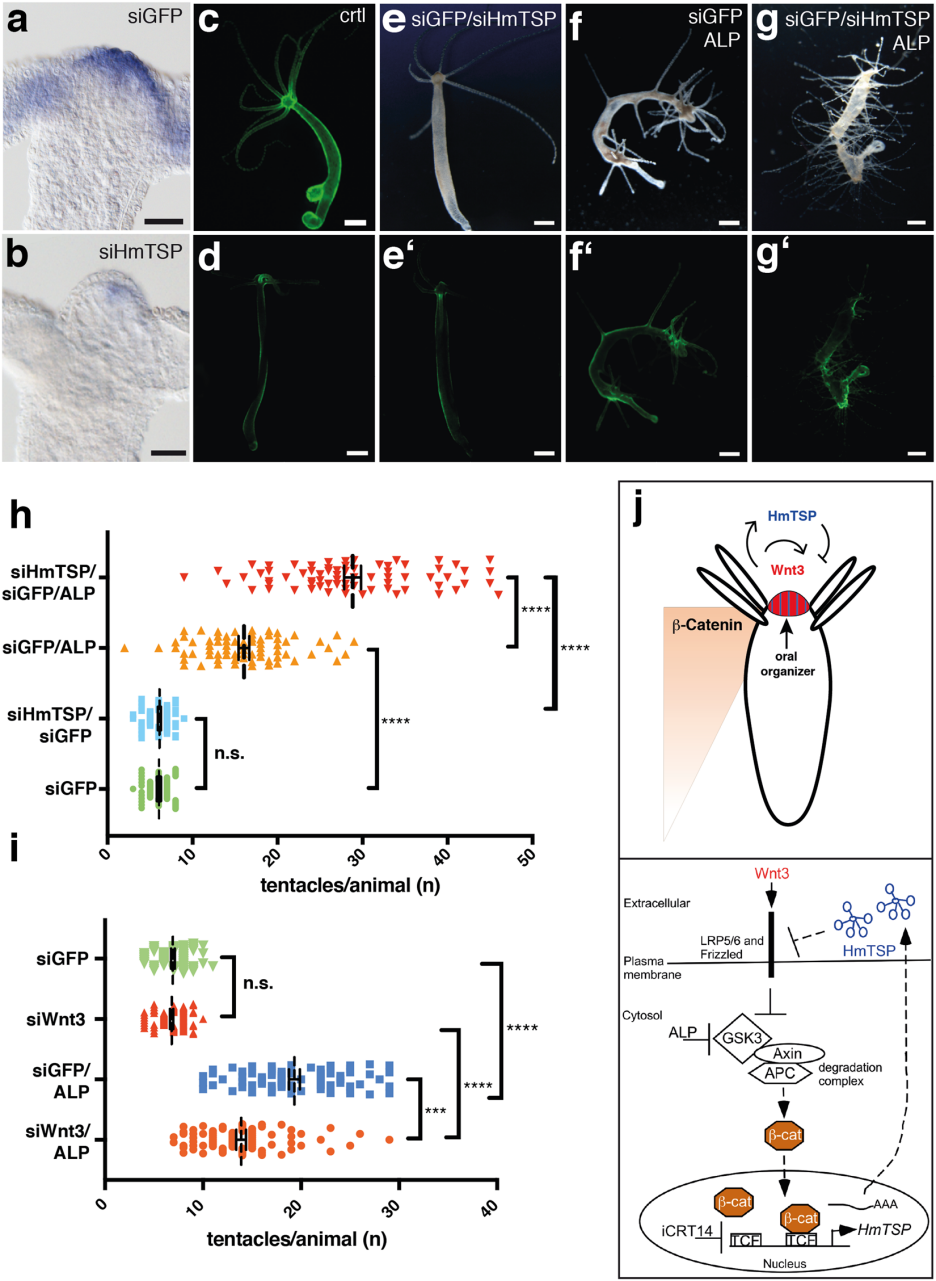
Functional analysis of *HmTSP* by siRNA knockdown. (**a**) ISH analysis of *HmTSP* expression in control *GFP*-transgenic animals electroporated with *GFP* siRNA. (**b**) ISH analysis showing reduced *HmTSP* expression in animals electroporated with *HmTSP* siRNA. Bars = 100 µm. (**c**,**d**) Demonstration of effective siRNA knockdown for *GFP* in transgenic hydra with ectodermal *GFP* expression. (**c**) Untreated control animal. D. Representative animal of the same transgenic strain as in C 8 days after electroporation with *GFP* siRNA. Bars = 500 µm. (**e–**e’) Treatment with combined siGFP and siHmTSP does not induce morphological changes in steady state polyps. (**f**,**g**) Treatment with ALP of siTSP electroporated animals resulted in a dramatic increase of ectopic tentacles compared to the siGFP-treated control group. f’ and g’ show the reduced *GFP* expression in the respective animals. Each panel representative of at least 10 hydras examined. (**h**) Quantification of ALP-induced ectopic tentacles in animals electroporated with siGFP or siGFP and siTSP. Animals electroporated with the respective siRNAs without subsequent ALP treatment served as controls. ALP treatment was performed 8 days after electroporation and the numbers of tentacles/animal in each group were counted 5 days after ALP treatment. Animals in d and e were recorded at the same time point after electroporation as animals in f and g. Animals (n) in each group were: siGFP = 67; siGFP/siTSP = 67; siGFP/ALP = 71; siGFP/siTSP/ALP = 71. (**i**) Effect of *Wnt3* depletion on ectopic tentacle formation: Animals were electroporated with siRNAs specific for *GFP* or directed against *Wnt3* and *GFP* followed by ALP- or control treatment as indicated. Ectopic tentacle formation was analyzed as described above (**h**). Number of polyps analyzed in each group was: siGFP: 74, siGFP/siWnt3: 76, siGFP/ALP: 79, and siGFP/siWnt3/ALP: 81. (**h** and **i** show the results from three independent experiments. Each data point represents a single hydra, bars indicate the mean ± S.E.M. ***P value < 0.0001; ns = not significant. The data were analyzed using the Kruskal-Wallis test followed by pairwise multiple comparison of each group with the other groups. (**j**) Model for the negative regulatory function of HmTSP on Wnt/β-Catenin signaling in the *Hydra* head organizer. Upper panel: *Wnt3* induces its own expression and that of *HmTSP*, the latter inhibits Wnt3 activity at the protein level thereby restricting β-Catenin activity to the head region. Lower panel: schematic diagram at the cellular level of *HmTSP* as a target of canonical Wnt signaling.

siRNA knockdown by electroporation has recently been established as a powerful tool for functional gene analysis in *Hydra*^29^. We tested the efficiency of a gene-specific *HmTSP* siRNA in electroporated animals using green fluorescent protein (*GFP)* siRNA as a control. ISH confirmed that the expression of *HmTSP* was markedly reduced in animals treated with *HmTSP* siRNA compared to *GFP* siRNA-treated controls (Fig. 8a,b). To visualize the systemic effect of siRNA transfection we used transgenic hydras that express *GFP* throughout the ectoderm^31,32^ (Fig. 8c). Transfection with siGFP resulted in a significant knockdown of *GFP* expression throughout the bodies of the electroporated *GFP*-transgenic animals. GFP depletion reached its full extent one week after electroporation and remained stable for another two weeks^33^, and the polyps retained the normal morphology of adult *Hydra* (Fig. 8d, and tentacles quantified in 8 h). This was also the case for animals electroporated with *HmTSP* and *GFP* siRNAs (Fig. 8e,e’,h) indicating that *HmTSP* depletion at the mRNA level does not affect patterning in steady state adult polyps.

To assess the role of *HmTSP* during organizer formation, we induced ectopic tentacles in *HmTSP* siRNA-treated animals by ALP treatment given 8 days after electroporation. Although animals treated with control siGFP and ALP increased tentacle numbers as expected (Fig. 8f,f’), hydras electroporated with the combination of *HmTSP* and *GFP* siRNAs and then treated with ALP showed a dramatic increase of ectopic tentacle formation compared to the siGFP control group without alteration in body size (Fig. 8g,g’). Quantification of ectopic tentacles in both of these groups at day 5 after ALP treatment demonstrated an almost two-fold difference in the mean tentacle number/animal (Fig. 8h). In addition, we noticed that ectopic tentacles appeared earlier in the *HmTSP* and *GFP* siRNA-treated group, than in the control siRNA-treated group. These data indicate that the knockdown of *HmTSP* expression rendered the epithelial tissue of the body column more permissive for the formation of ectopic signaling centers. Although *Wnt3* knockdown did not affect tentacle numbers, interestingly, a siRNA knockdown of *Wnt3* with subsequent ALP treatment reduced ectopic tentacle numbers as compared to the siGFP/ALP control (Fig. 8i), suggesting that our experiment likely addresses an autocatalytic maintenance of the organizer after removal of the GSK3-β inhibitor.

## Discussion

Self-regulation by diffusible morphogens and their antagonists is a key feature of so-called morphogenetic fields in the developing embryo of metazoans^34^. Wnt/β-Catenin signaling has a major role in embryonic axis determination and anterior-posterior (A-P) patterning. To date, there is very limited knowledge of the associations of ECM components with this process. Matrix metalloproteases, such as Tolloid, degrade Chordin and promote BMP signaling in the patterning of the dorso-ventral axis^35^. Drosophila homologues of the SMOC and SPOCK matricellular proteins, magu, and cow, respectively, act in the wing imaginal disc to down-regulate Wnt signaling in the case of magu or extend the range of the gradient, in the case of cow^36,37^. Recently, glypican, a cell-surface heparan sulfate proteoglycan, was characterized as a critical factor in negative feedback of canonical Wnt signaling by acting as a scaffold that co-localizes Wnt ligands and the Wnt-specific deacetylase Notum^38^.

To our knowledge, our data provide the first evidence for functional association of a TSP with Wnt signaling *in vivo*. To date, TSPs have been studied most intensively in mammals, where they constitute a five-gene family of trimeric (TSP1, TSP2) and pentameric (TSP3–5) forms. Gene knockout mice for each of the five TSPs are viable and have revealed important post-developmental and tissue-specific roles for individual TSPs, especially in the cardiovascular system, skeletal muscle and tendons, synaptogenesis and immune cell functions^15,39,40^. The underlying mechanisms relate to interactions of TSPs at cell-surfaces, with proteases, cytokines, or within the ECM. Multiple morphogens also have post-developmental roles in mammalian tissues, stem cell niches or disease pathologies^41,42^. Indeed, a few instances of modulation of morphogen signaling by TSPs have been identified: promotion of Notch signaling by TSP2 or TSP4^43,44^, or antagonism of VEGF and VEGF-receptor 2 by TSP1 and TSP2^45^. Wnt family members also have many post-developmental functions in mammals, for example in cartilage differentiation, homeostasis and osteoarthritis^46^, and in control of the intestinal stem cell niche^47^. Aberrant activation of the Wnt pathway is associated with cancer and other diseases^48,49^. Although TSP family members have known roles in these contexts, the possibility of functional or regulatory links between TSPs and Wnts has received little attention. Circumstantially, the absence of TSP1 in a mouse experimental model of colorectal cancer correlated with up-regulation of transcripts encoding several negative regulators of Wnt signaling^50^. A Wnt antagonist, secreted frizzled-related protein 1 (sFRP1) has been found to bind the *N*-terminal domain of TSP1 *in vitro* but the physiological relevance of the interaction remains unknown^51^.

In this regard, it can also be acknowledged that, despite major advances from the study of TSP gene knockout mice, functional redundancy in the context of a gene family with over-lapping tissue expression patterns of the family members presents an inherent difficulty to understanding the fundamental roles of TSPs in mammals. This complexity has heightened interest in invertebrate models, many of which encode a single TSP^11^. Studies of the single pentameric TSP of *Drosophila melanogaster* demonstrated its importance during development for ECM organization and integrin-binding at muscle-tendon attachment sites^52–54^. However, the possibility to compare the roles of TSP between *Drosophila* and mammals is limited fundamentally by the absence of fibrillar collagens from *D. melanogaster*^55^. Fibrillar collagens are the most abundant components in mammalian connective tissue ECM and appear very relevant to the functions of TSPs. All TSP gene knockout mice studied to date exhibit aberrant collagen fibril morphology and packing in certain tissues, that correlate in several cases with mechanical weakening of the tissue^20,56–59^, and also, specific binding sites for TSP1 have been identified in collagens I–III^60^. *Hydra* is therefore of great interest as an invertebrate model because of the closer relationship of cnidarian mesoglea composition and organization to the ECM structures of mammals^6^.

Our analysis of the domain and oligomerization properties of HmTSP demonstrated many similarities with the pentameric TSPs of vertebrates. Like HmTSP, these TSPs lack thrombospondin type 1 (TSR) domains. A *Hydra* protein with TSR domains previously annotated as “Hydra TSP” in fact lacks the C-terminal domains characteristic of TSPs and corresponds to a TSR superfamily member^61,62^. The absence of TSR-containing TSPs in cnidarians has been corroborated by a large-scale study of TSR proteins, none of which correspond to the TSP domain architecture^63^. Several transcripts encoding TSR-proteins are expressed in the region of the hypostome^64^ and others are involved in cnidarian-dinoflagellate symbiosis^63^. However, these TSR proteins were not identified as part of the mesoglea proteome by our study. Notwithstanding the weak coiled-coil of HmTSP, ectopically expressed intact HmTSP protein, or a truncated *N*-terminal protein including the predicted ‘oligomerization region’ are secreted as pentamers. Whereas synthetic versions of the coiled-coil domain of mammalian TSPs have independent pentamerization (shown for TSP5)^65^ or trimerization (shown in this study for TSP1) activity, the ‘oligomerization region’ of HmTSP self-assembled as a trimer and had low thermal stability. We infer that, unlike mammalian TSPs, the adjacent paired cysteines have a major role in stabilizing pentamer assembly. Analysis of a set of cnidarian TSP sequences established that the absence of a coiled-coil domain is not a universal property of cnidarian TSPs, because *A. digitifera* TSP includes a region with strong coiled-coil probability. However, whether the coiled-coil domain of TSPs originated in cnidarians, or alternatively, has undergone rapid evolutionary divergence in multiple cnidarian lineages, cannot be determined without further study of TSPs in other phyla of early-diverging metazoans. Bilaterians have evolved a variety of oligomerization states of TSPs apparently through domain shuffling that affects the *N*-terminal region of TSPs^11,16^.

In *Hydra*, the hypostome constitutes a blastopore-like organizer, where canonical Wnt signaling factors regulate the process of anterior-posterior axis determination during steady state and head regeneration^24,66,67^. Different from higher metazoans, in which axial patterning is restricted to embryogenesis, the high tissue dynamics of *Hydra* require a constant activity of Wnt factors in the head region^66,68^. Self-regulation of the Wnt signaling center at the transcriptional level was demonstrated previously by a functional analysis of the *Hydra Wnt3* promoter. In addition to a positive feedback loop via β-Catenin/TCF, this approach revealed a negative regulatory sequence element that restricts *HyWnt3* expression to the apical hypostome area^30^. Our *in situ* hybridization data now demonstrate a similar restricted expression of *HmTSP*, which contrasts with the widespread expression of transcripts for other prominent mesoglea components such as laminin or collagen chains^7,9,10,21^.

Our data indicate that *Hydra* thrombospondin acts at the protein level in a negative feedback loop of β-Catenin signaling, in direct contact to *Wnt* expressing cells of the hypostome. Our results also establish direct positive regulation of *HmTSP* expression by β-Catenin/TCF and demonstrate that the axis-inducing capacity of the epidermal tissue is increased when *HmTSP* expression is diminished. We therefore propose that HmTSP has a negative regulatory function in the mesoglea that limits high canonical β-Catenin activity to the hypostomal organizer (Fig. 8j).

These findings are in line with the pattern forming system proposed by Meinhardt and Gierer^69^ that in its simplest form consists of an autocatalytic activator (Wnt) that induces the production of its own inhibitor (Fig. 8j). We have to emphasize, though, that our data indicate a modulatory effect on Wnt activity rather than complete inhibition. Although siRNA-silencing of *HmTSP* strongly reduced transcript levels, phenotypic consequences for hypostome function were manifested only when animals were challenged with ALP. This might indicate a greater stability of HmTSP protein at the pre-existing natural hypostome, which is in line with the observation of Aufschnaiter *et al*. that the mesoglea of the head region is more stationary compared to the body^13^. Alternatively, more overt consequences of the *HmTSP* knockdown might be detected in progeny generations. The mechanism of Wnt inhibition by HmTSP remains to be analyzed but could involve effects on Wnt ligand or receptor mobility or turnover. It should be emphasized that the thrombospondin type 1 domains by which R-spondins enhance Wnt signaling^70^ are not present in HmTSP. HmTSP might bind Wnt or a secreted Wnt inhibitor or could affect ECM properties (for example through collagen-binding) to indirectly limit the diffusability of Wnt ligand. An interesting alternative mechanism of restriction could be to hold Wnts (as fatty-acid modified proteins), in a lipophilic inner pore formed by the pentamerizing oligomerization domain; in TSP5 this domain has binding capacities for hydrophobic molecules^18,71^. Given the known effects of mammalian TSPs on Notch endocytosis or VEGF receptor trafficking, effects on cell surface-exposure or functions of frizzled proteins or co-receptors are also an obvious possibility.

## Materials and Methods

### Materials

Chemicals were obtained from Sigma-Aldrich (Merck, Darmstadt, Germany) unless otherwise indicated. COS7 green monkey kidney cells (ATCC, Manassas, VA, USA: CRL-1651) were maintained in DMEM with 10% fetal calf serum (FCS). HEK293T human embryonic kidney cells **(**DSMZ, Braunschweig, Germany: ACC635) were maintained in the same medium containing 100 U/mL penicillin and 100 µg/mL streptomycin (ThermoFischer Scientific, Waltham, MA, USA). All cells were maintained in a humidified 5% CO_2_ atmosphere at 37 °C. Recombinantly expressed TSP proteins were detected using a mouse monoclonal penta-His-antibody (Qiagen, Hilden, Germany: 34660) or a mouse monoclonal antibody directed against the V5-tag (Invitrogen, Thermo Fisher Scientific, Waltham, MA, USA: R960-25). PAGE protein markers used included Precision Plus dual color standards (Bio-Rad, Munich, Germany), PageRuler prestained protein ladder (Thermo Fisher Scientific), or HiMark™ pre-stained high molecular weight protein standard (Invitrogen). Monoclonal antibody mAb52 to hydra laminin^72,73^ was a kind gift of Xiaoming Zhang.

### Animals

*Hydra magnipapillata* strain 105 was used for all experiments, except for siRNA treatment. For siRNA experiments, a transgenic *Hydra vulgaris* strain expressing ectodermal GFP and endodermal RFP provided by Robert Steele’s laboratory was used^31^. Nucleotide sequence identity of *TSP* between these species is 99.9%. All animals were maintained in artificial *Hydra* medium (HM, 1 mM CaCl_2_, 0.1 mM MgCl_2_, 0.1 mM KCl, 1 mM NaH_2_CO_3_, pH 6.8) at 18 °C in polystyrene dishes (Carl Roth, Karlsruhe, Germany) and fed two to three times per week with freshly hatched *Artemia salina* nauplii, unless indicated otherwise. Media was renewed 3–4 hrs after feeding and again the following day. Animals were starved for 24 hrs prior to experiments.

### Mesoglea Extraction and Mass Spectrometry Analysis

100 normal-sized hydras without buds were collected into several 1.5 ml Eppendorf tubes, washed in deionized water and pelleted for 10 min at 13,000 rpm. The supernatant was discarded and 500 µl 0.5% N-lauroylsarcosinate solution was added to the animals. Samples were snap-frozen in liquid nitrogen and thawed at RT resulting in detachment of the epithelia from the mesoglea. The mesoglea was washed 5–10 times in fresh deionized water to remove cellular material and the purity of the isolated mesoglea was confirmed by light microscopy. For laminin staining of isolated mesoglea the monoclonal mab52 antibody was used at 1:1000. Mesoglea samples were dissolved in 1x NuPAGE® lithium dodecyl sulfate (LDS) buffer (Thermo Fisher Scientific) containing 1 M DTT, boiled at 90 °C for 30 min and loaded after a quick spin onto a NuPAGE® Bis-Tris 4–12% gel (Thermo Fisher Scientific). The gel was stained with colloidal Coomassie (Bio-Rad) and the lane was cut into 27 pieces. In-gel tryptic digestion and extraction was performed as previously described^74^. Peptide separation was achieved using a nano Acquity UPLC system (Waters, Eschborn, Germany). The nano UPLC system was coupled online to an LTQ OrbitrapXL mass spectrometer (Thermo Fisher Scientific). Data dependent acquisition with Xcalibur 2.0.6 (Thermo Fisher Scientific) was performed by one FTMS scan with a resolution of 60000 and a range from 370 to 2000 m/z in parallel with six MS/MS scans of the most intense precursor ions in the ion trap. The mgf-files were used for database searches with the MASCOT search engine (Matrix Science, London, UK) against *Hydra* Hma2 Protein models and GenBank Proteins. Domain composition of GenBank protein accessions was analyzed against CDD (NCBI) and InterProScan 5 (EMBL-EBI).

### cDNA Cloning

RNA was isolated with the RNAeasy Kit (Qiagen). Around 100 *Hydra magnipapillata* were collected in a 1.5 ml Eppendorf tube, the remaining HM discarded and 350 µl RNeasy lysis buffer with freshly admixed ß-mercaptoethanol (1% v/v) added. Further processing was according to manufacturer’s instructions. cDNA synthesis was performed with SuperScript III reverse transcriptase kit (Invitrogen) according to manufacturer’s instructions. PCR amplification of *HmTSP* was performed using PRECISOR high fidelity DNA polymerase (BioCat, Heidelberg, Germany) according to manufacturer’s instructions on 10 ng of cDNA template and with the gene specific primers *HmTSP*forward: 5′AAACTTTTTAGTCCCTGTGTAATAAG and *HmTSP*reverse: 5′TTCATACATTTCTAATTTTGTGTCA. The PCR product was purified (Wizard^®^ SV gel and PCR clean-up system, Promega, Mannheim, Germany) and additional Poly-A tailing was performed. The purified PCR product was ligated into the pGEM-T plasmid (Promega) and the DNA sequence determined by automated sequencing (Eurofins-MWG Europe, Ebersberg, Germany) using T7 sense, SP6 anti-sense, and HmTSP-specific primers. A partial HmTSP cDNA was also sequenced from pBS.SK encoding GenBank CN557414 (a gift from Hans Bode, UC-Irvine) using T3 and T7 primers.

### Real-time Quantitative PCR

To examine *HmTSP* expression *in vivo* (distribution analysis in Fig. 4d), 25 budless animals were cut at 50% body length and the upper (head) and lower body tissues were instantly transferred into TRIzol^®^ reagent (Life Technologies, Thermo Fisher Scientific) and stored at −20 °C. For regeneration experiments, 50 budless animals were cut at 50% body length. Upper and lower tissues were transferred into petri dishes containing fresh HM. At specific time points the regenerating tips (10% of body length) were cut off and immediately transferred into TRIzol^®^ and stored at −20 °C. For isolation of RNA from siRNA electroporated animals ten whole animals of each condition were collected into TRIzole and store at −20 °C until further use. RNA isolation and transcription into cDNA was performed according to manufacturer’s instructions (SensiFAST™ cDNA synthesis kit, Bioline, London, UK) and quantitative real-time PCR was carried out with PCR System StepOnePlus (Applied Biosystems, Thermo Fisher Scientific) and SensiFAST SYBR Hi-ROX Kit (Bioline, London, UK). Transcript levels were quantified by the comparative C_t_ Method. Three biological replicates were performed for each experiment and triplicate measurements were made for each sample in each experiment. No template conditions served as negative controls. The housekeeping gene *γ-tubulin* was used to normalize the data. The qPCR primer sequences are given in Supplementary Table S2.

### *In situ* Hybridization

To generate RNA probes, a partial *HmTSP* cDNA from GenBank EST sequence CN557414 (bp 1022–2475 + 487 bp of 3′ untranslated region in pBS.SK) was used. A plasmid template for the *HmWnt3*-specific RNA probe was described recently^75^. For generation of GFP-specific probes, a 673 bp fragment of the GFP-coding sequence was amplified on cDNA from GFP expressing transgenic *Hydra* by PCR using primers GFPfw (5′-GGAGAAGAACTTTTCACTGGAGTTGTCC-3′) and GFPrv (5´-CAGCAGCTGTTACAAACTCAAGAAGGACC-3′), and subcloned into pGEM-T easy. Digoxygenin-labeled RNA probes for *HmTSP*, *HmWnt3*, and *GFP* fluorescein isothiocyanate (FITC)-labeled RNA probes corresponding to the sense and antisense strands were prepared using an RNA labeling *in vitro* transcription kit (Roche, Basel, Switzerland). Animals were relaxed in 2% urethane for 1–2 min in HM and fixed overnight at 4 °C in 4% formaldehyde and cleared in two changes of 100% methanol for 10 min each and stored at −20 °C until further use. To block endogenous peroxidase cleared animals were incubated for 30 min in methanol containing 1% hydrogen peroxide. Animals were then rehydrated through a grades series of methanol (100%, 75%, 50%, and 25%) in PBT (phosphate-buffered saline with 0.1% Tween-20) for 5 min each and rinsed three times for 5 min each in PBT. After digestion for 7 min with 10 mg/ml proteinase K in PBT, animals were washed in 4 mg/ml glycine in PBT for ten min, the glycine removed by two 5-min washes in PBT, and samples treated twice for 5 min with 0.1 M triethanolamine (TEA) and 5 min with 0.25% (v/v) acetic anhydride in 0.1 M TEA, followed by 0.5% (v/v) acetic anhydride in 0.1 M TEA. After two further washes in PBT, samples were fixed for 20 min in 4% formaldehyde in HM at room temperature, then washed five times for 5 min in PBT. Samples were washed for 10 min in 50% PBT/50% hybridization solution (HS, 1:1 mixture of deionized formamide and buffer containing 5x SSC (750 mM NaCl, 75 mM sodium citrate), 0.2 mg/ml yeast tRNA, 2% of 50x Denhardt’s solution, 0.1 mg/ml heparin, 0.1% Tween-20 and 0.1% CHAPS), then incubated for 2 hrs in HS at 55 °C. Digoxygenin-labeled *HmTSP* RNA probes or combinations of digoxygenin-labeld *HmWnt3* or *GFP* together with FITC-labeled *HmTSP* RNA probes (dual color fluorescent *in situ* hybridizations) were added to a final concentration of 0.05 ng/µl in fresh HS and hybridized for 60 hrs at 55 °C. Unbound probe was removed by washing for 5 min each in 75% HS/25% 2x SSC; 50% HS/50% 2x SSC, and 25% HS/75% 2x SSC, at 55 °C, and the samples washed twice for 30 min each in 2x SSC with 0.1% CHAPS at 55 °C. Next, samples were washed twice for 10 min at room temperature in MAB (100 mM maleic acid, 150 mM NaCl, pH 7.5) and blocked for 2 hrs in blocking solution (BS, MAB containing 1% blocking reagent (Roche, Basel, Switzerland). For colorimetric *in situ* hybridizations, samples were incubated overnight at 4 °C in BS containing alkaline phosphatase-conjugated anti-digoxygenin Fab fragments (1:4000, Roche: 11093274910). Unbound Fab fragments were removed by eight 30 min washes in MAB at room temperature. Samples were then equilibrated with alkaline phosphatase staining buffer NTMT (100 mM NaCl, 100 mM Tris, pH 9.5, 50 mM MgCl_2_, 0.1% Tween-20) by two 5–10 min washes. NTMT buffer was removed and 300 µl BM Purple (Roche) substrate solution was added to the samples followed by incubation in the dark for 30 min at 37 °C. Staining was stopped by two 10 min washes in absolute ethanol and three PBS rinses. In case of dual color sequential detection of FITC- and digoxygenin-labeled RNA-probes the TSA Plus Cyanine 3 and Cyanine 5 System (PerkinElmer, Waltham, MA, USA) was used according to the manufacturer’s instructions. Samples were mounted in 90% glycerol in PBS or in Mowiol 4–88 (Carl Roth).

### Alsterpaullone and iCRT14 Treatment

ALP treatment was performed as previously described^27^. ALP was dissolved in DMSO at a stock concentration of 25 mM and aliquots were stored at −80 °C in the dark. An aliquot of the stock solution was diluted to 2.5 µM final concentration in HM immediately prior to use. Control animals were, in parallel, treated with 0.01% DMSO. iCRT14 is a potent inhibitor of β-catenin-responsive transcription in assays of Wnt pathway activity^76^. To inhibit ß-catenin, budless hydra polyps were incubated with 50 µM iCRT14 in HM. After 24 hrs chemicals were washed out by rinsing twice with HM, polyps incubated for 72 hrs in HM, and fixed for *in situ* hybridization.

### Electroporation with siRNAs

siRNAs (HPLC grade) specific for GFP and HmTSP (siGFP, scrambled siGFP, siTSP1 and siTSP2, see Supplementary Table S2 for sequences) were purchased from Qiagen. Electroporation of siRNA was performed as described recently^33^ using 3 µM of siGFP (1 µM siGFP and 2 µM scrambled siGFP) or a combination of siGFP, siTSP1, and siTSP2 (1 µM each). In brief, animals from a daily-fed culture of chimeric ecto-GFP/endo-RFP transgenic animals^32^ were washed twice with ultrapure water. For each reaction, 20 animals were transferred to electroporation cuvettes (4 mm gap, Bio-Rad) and excess liquid was removed. After adding 200 µl of sterile ultrapure water containing either 3 µM of siGFP (1 µM siGFP and 2 µM scrambled siGFP) or a combination of siGFP, siTSP1, and siTSP2 (1 µM each) animals were allowed to relax for 20 min at room temperature. Thereafter, a single square pulse at 250 V was administered for 25 ms using a GenePulser Xcell^TM^ (Bio-Rad) electroporation system equipped with a CE module. Immediately after the pulse, 500 µl restoration medium consisting of 80% HM and 20% hyperosmotic dissociation medium (6 mM CaCl_2_, 1.2 mM MgSO_4_, 3.6 mM KCl, 12.5 mM TES, 6 mM sodium pyruvate, 6 mM sodium citrate, 6 mM glucose and 50 mg/l rifampicin, 100 mg/l streptomycin, 50 mg/l kanamycin, pH 6.9) was added to the cuvette, the animals were transferred to Petri dishes containing restoration medium and allowed to recover for one day. Viable polyps were transferred to new dishes containing HM and maintained under standard culture conditions. For ALP treatment, animals were incubated at 8 days post-electroporation either with 0.1% DMSO or 2.5 µM ALP in HM for 16 hrs. Thereafter, they were rinsed in HM several times and cultured under standard conditions. 5 days after ALP treatment, the animals were fixed with 4% formaldehyde and the total number of tentacles was counted by placing a single hydra per petri dish and turning them once with forceps. Images were taken using a Nikon SMZ25 stereomicroscope equipped with Nikon DS-Ri2 high-definition color camera.

### Chromatin immunoprecipitation

Chromatin immunoprecipitation (ChIP) analysis was carried out as described recently by using sheared extracts from formaldehyde-treated animals, and an antiserum directed against a recombinant *Hydra* TCF protein^26^. PCR of precipitated DNA was carried out using specific primers flanking the potential TCF binding sites or a control region in the 5′-regulatory region of the *HmTSP* gene (Fig. 7a) using 54 °C as primer annealing temperature. PCR primer sequences are given in Supplementary Table S2.

### Microscopy and Digital Image Processing

Images were acquired with a Nikon Digital Sight DS-U1 camera mounted on Nikon Eclipse 80I and imaging software NIS Elements (3.10, SP3, Hotfix, Build645). Further image processing was performed with Adobe Photoshop CS6 and Fiji.

### Peptide Synthesis

Rink amide ChemMatrix resin (PCAS Biomatrix, Quebec, Canada) and fluorenylmethoxycarbonyl (Fmoc)-L-amino acids derivatives were from AGTC Bioproducts (Hessle, UK). 2-(1H-benzotriazol-1-yl)−1,1,3,3- tetramethyluronium hexafluorophosphate (HBTU) was from GL Biochem (Shanghai, China). All other reagents were of peptide synthesis grade and were from Thermo Fisher Scientific (Loughborough, UK). HsTSP1cc (43-mer) and HmTSPcc (25-mer) peptides (sequences shown in Supplementary Fig. S1) were synthesized at 0.1 mmol scale on Rink amide resin in a Liberty 12 microwave peptide synthesizer (CEM Corporation, Buckingham, UK) using standard Fmoc solid-phase methodology^77^. HsTSP1cc peptide was synthesized in several stages to avoid unwanted interactions between hydrophobic residues prior to complete synthesis. First, EENKELANELRRGW peptide was synthesized by automated procedure. Next, VT-pseudoproline was added manually to the initial sequence to minimize aggregation between hydrophobic residues. 2.5 equivalents of VT-pseudoproline, 2.4 equivalents O-(7-azabenzotriazol-1-yl)-N,N,N′,N′-tetramethyluronium hexafluorophosphate and 3 equivalents of diisopropylethylamine were dissolved in 5 ml of N-Methyl-2-pyrrolidone (AGTC Bioproducts, Hessle, UK). The mixture was incubated on a rotating wheel for 2 hrs at 20 °C, then washed 3 times with 5 ml of dimethylformamide (DMF) followed by 5 ml of dichloromethane (DCM). Next, the IRK sequence was added by automated synthesis. DS-pseudoproline was then added manually as described above. Finally, the remaining amino acids, GDELSSMVLELRGLRTIVTTLQ, were added by automated synthesis. For each automated step, Fmoc amino acid (5 equivalents), HBTU activator (4.5 equivalents) and DIPEA (10 equivalents) were mixed in 7 ml of DMF for 5 min with 20-watt microwave irradiation at 75 °C. For deprotection, peptides were incubated in 20% piperidine in DMF for 5 min with 20-watt microwave irradiation at 75 °C. After automated linear assembly, the peptide was manually *N*-terminally acetylated using acetic anhydride (3 equivalents) and pyridine (4.5 equivalents) in DMF (7 ml) for 30 min. HsTSP-1cc peptide was cleaved manually from the resin using a mixture of 95:5:5 trifluoroacetic acid (TFA), triisopropylsilane and water in a total volume of 10 ml for 2 hrs at 20 °C. After washing with an additional 5 ml of TFA, acid extracts were combined and concentrated to a final volume of 5 ml under nitrogen flow. Crude peptides were precipitated in ice-cold diethyl ether (40 ml) and isolated by centrifugation at 4000 rpm for 15 min at 2 °C. Finally, each pellet was re-dissolved in a total volume of 10 ml acetonitrile (MeCN)/water 1:1 (v/v) and freeze-dried.

### Peptide Purification and Identification

Peptides were purified by reverse phase-high performance liquid chromatography (RP-HPLC; JASCO, Dunmow, UK) using a Kromatek C18 reverse phase column (10 mm inner diameter x 150 mm long). 5 mg of each crude peptide was dissolved in water, centrifuged for 5 min and injected into RP-HPLC. The following eluents were used: A) 0.1% TFA in water, B) 0.1% TFA in MeCN. Each peptide was eluted by running a linear gradient of 30% to 70% (HsTSP-1cc) or 20% to 80% (HmTSPcc) MeCN in water at a rate of 3 ml/min over 30 min. Fractions were collected around the peak of peptide elution, as detected by UV absorption at 280 nm. Fractions were analyzed by MALDI time-of-flight (TOF) mass spectrometry (Applied Biosystems 4700 Proteomics Analyzer MALDI instrument, Thermo Fisher Scientific). Only fractions found to contain peptide exclusively were pooled and lyophilized. To confirm the purity of each peptide by analytical RP-HPLC, a small amount of peptide was dissolved in water and run on a gradient of 20% to 80% MeCN for 20 min (mass spectrometry and HPLC data are show in Supplementary Fig. S1).

### Circular Dichroism Spectroscopy

Circular dichroism (CD) measurements were conducted using a JASCO J-815 spectropolarimeter fitted with a Peltier temperature controller (Jasco) according to previously described methods^78^. The concentration of HsTSP1cc peptide was measured by UV absorption at 280 nm according to the Beer-Lambert equation (ε(Trp) = 5690 mol^−1^ cm^−1^), where ε is the molar absorptivity of tryptophan. For experiments, HsTSP1cc was dissolved in PBS (8.2 mM sodium phosphate, 1.8 mM potassium phosphate, 137 mM sodium chloride, 2.7 mM potassium chloride, pH 7.4) at two different concentrations, 10 μM and 100 μM, and HmTSPcc was dissolved in PBS at 100 μM. Full CD spectra were recorded from 260 nm to 196 nm in 2 mm and 1 mm path-length quartz cuvettes at 5 °C. The instrument was set with a scan rate of 100 nm/min, a 1 nm interval, a 1 nm bandwidth and a 16 second response time. The data collected were converted from ellipticities (millidegrees) to mean residue ellipticity (MRE: deg cm2/dmol res) by normalizing for amide bond concentration and cuvette path length.

### Analytical Ultracentrifugation

Sedimentation equilibrium experiments were performed at 20 °C in a Beckman-Optima XL-I analytical ultracentrifuge using an An-60 Ti rotor (Beckman-Coulter, High Wycombe, UK). Peptides were dissolved in PBS at 50 μM (HsTSP1cc) or 400 µM (HmTSPcc); the higher concentration of the latter was used to ensure the folding of HmTSPcc. Peptide was centrifuged at speeds in the range 20,000–50,000 rpm (HsTSP1cc) or 30,000–46,000 rpm (HmTSPcc). Collected data sets were fitted to a single, ideal species model using Ultrascan (http://www.ultrascan.uthscsa.edu/). Since the data fitted well to the single-ideal species model, more complex models were not used.

### Secretion and Oligomerization of *Hydra* TSP in Mammalian Cells

The mammalian expression plasmids for human TSP5.V5 and TSP1.V5 have been described previously^20,79^. cDNAs encoding full-length *Hydra* TSP was amplified by PCR using the primer pair 740 F/742 R (740 F, 5′CCTGATTACAAGGGATGCTTC and 742 R, 5′CTTCTTGAACTTAGAATTGTAGTCTTC). The *N*-terminal (N) and coiled-coil oligomerisation domain (o) only (referred to as “NoHmTSP”) was PCR amplified with primer pair 740 F and 743 R (743 R, 5′ACCAGGGGATTCAGGTCCTTC). PCR products were resolved on agarose gels, and DNA purified and ligated into pcDNA3.1V56His.TOPO (Invitrogen) according to manufacturer’s instructions. DNA sequences were confirmed by automated DNA sequencing (Eurofins-MWG Europe). Plasmids were transfected into COS7 cells plated at 8 × 10^5^ cells/90 mm dish or 2.5 × 10^5^ cell/60 mm dish with Polyfect (Qiagen, Manchester, UK), according to manufacturer’s instructions. 42 hrs later, conditioned media were collected, centrifuged at 1200 rpm to remove cells and Halt EDTA-free protease inhibitor (Thermo Fisher Scientific) added. TSP proteins were collected onto Talon metal affinity resin (Clontech, Saint-Germain-en-Laye, France) by incubation with rotation at 4 °C for 2 hrs. Beads were washed 3 times in TBS containing 2 mM CaCl_2_ and Halt protease inhibitor (Thermo Fisher Scientific). Bead-bound proteins were released by boiling in SDS-PAGE sample buffer and resolved on 7% SDS-polyacrylamide gels (SDS-PAGE), in the absence or presence of 100 mM dithiothreitol (DTT) as reducing agent. For resolution of non-reduced NoHmTSP protein, or full-length HmTSP, gels were run until the 75-kDa marker was near the bottom of the gel. Proteins were transferred to Immobilon-P PVDF membrane (Merck-Millipore, Darmstadt, Germany) by semi-dry electrophoresis (Bio-Rad, Watford, UK). Membranes were blocked for 16 hrs in TBS containing 2% powdered milk and 0.2% Tween-20 buffer, incubated with V5 antibody for 2 hrs, washed 3 times over 30 min in blocking buffer, incubated with goat-anti-mouse IgG-AP (Applied Biosystems, Thermo Fisher Scientific) for 1 hr, washed again 3 times for 10 min each, washed twice in PBS for 5 min each, twice for 5 min in 1 mM MgCl_2_ containing 1% diethanolamine and then incubated in 0.5% CSPD (Applied Biosystems, Thermo Fisher Scientific) in the same buffer for 5 min. Membranes were exposed to Hyperfilm ECL (Amersham, GE Healthcare, Little Chalfont, UK) and developed in an AGFA Curix X-ray film processor.

For expression of HmTSP in HEK293T cells, full-length HmTSP was subcloned into the pCEP-Pu mammalian expression vector, which contains a BM-40 signal sequence and an *N*-terminal histidine tag^80^. Cells were transfected with the HmTSP expression vector or pCEP-PU using TransIT^®^-LT1 Reagent (Mirus Bio LLC, Madison, WI, USA) according to the manufacturer´s instructions. After 48 hrs transfected cells were selected for 8 days in medium containing 1 µg/mL puromycin. For purification of recombinant HmTSP cells from two culture dishes (9 cm diameter) of HmTSP expressing or pCEP-PU transfected cells at 80% confluency were solubilized in 1 ml PBS containing 1% Triton X-100 and cOmplete^TM^ Protease Inhibitor Cocktail (Roche) for 1 hr at 4 °C with mild agitation. Non-solubilized material was removed by centrifugation at 2500 × g for 15 min at 4 °C. The resulting supernatant was adjusted to 10 ml PBS containing 0.1% Triton X-100 and 5 mM imidazole, combined with 100 µl of Amintra^TM^ Ni-NTA affinity resin (Expedeon Inc., San Diego, CA, USA), and incubated for 2 hrs at 4 °C with mild agitation. After binding, the resin was washed five times with 10 column volumes of PBS containing 0.1% Triton X-100 and 10 mM imidazole. Bound proteins were eluted with 200 µl non-reducing SDS-sample buffer at 95 °C for 10 min. Non-reduced samples were used directly and reduced samples were adjusted to 0.1 M DTT before SDS-PAGE analysis.

For SDS electrophoresis of high molecular weight HmTSP oligomers, precast 4–15% or 4–20% gradient gels (Bio-Rad) were used. For Western blotting, proteins were transferred in blotting buffer (0.1% SDS, 0.025 M TRIS, 0.192 M glycine, 20% v/v methanol) and the membrane was blocked in 5% milk powder, 0.1% PBS Tween-20 at RT for 1 h. Anti-penta-HIS antibody was diluted at 1:1000 in 0.5% milk powder containing 0.1% PBS Tween-20, and membranes were incubated overnight at 4 °C. Anti-mouse IgG horseradish peroxidase-conjugated antibody was applied at 1:10.000 and blots were developed using a peroxidase substrate for enhanced chemiluminescence.

### Bioinformatics and Molecular Phylogeny

Additional cnidarian TSPs were identified by BLAST searches of GenBank Protein or Nucleotide databases or the Transcriptome shotgun assembly (TSA) at NCBI, or through the resources of Compagen^81^, using human TSP-1 (NP_003237.2), human TSP3 (NP_009043.1) or *Hydra* TSP protein sequences as the search queries. Full-length cnidarian TSPs were identified from the transcriptomes of *Aurelia aurita*^82^ (GenBank TSA GBRG01126972.1), *Anthopleura elegantissima*^83^ (TSA GBXJ01117552), *Acropora millepora*^84^ (TSA JR974454), *Aiptasia pallida*^85^ (TSA JV114025.1), *Hydractinia symbiolongicarpus*^86^ (TSA GAWH01034818), *Porites australiensis*^87^ (TSA FX452258.1), and *Stylophora pistillata*^88^ (GARY01004999). Coiled-coil regions were predicted in MARCOIL^16,89^. Protein domain architectures were analyzed in InterProScan 5.0 at EBI^90^. Multiple sequence alignments (MSA) were prepared in MUSCLE 3.8^91^. The dataset of TSPs for phylogenetic analysis was based on that in^11^, with inclusion of TSP *C*-terminal regions from *Amphimedon queenslandica*^92^, *Nematostella vectensis*^17^, Oscarella carmela (Compagen), and cnidarian species as listed above. The region from the last EGF domain to the *C*-terminus of each TSP was used for multiple sequence alignment in MUSCLE 3.8 at default parameters followed by curation with gap removal. Phylogenetic analysis was carried out in PhyML 3.0^93^ at default parameters with 200 cycles of boot-strapping and tree rendering in iTOL^94^.

### Data availability statement

The datasets generated during and/or analyzed during the current study are available from the corresponding authors on reasonable request.

## Electronic supplementary material

Supplementary Information
