# Supplementary material for: Hydra Mesoglea Proteome Identifies Thrombospondin as a Conserved Component Active in Head Organizer Restriction
Source: Sci Rep. 2018 Aug 6;8:11753. doi: 10.1038/s41598-018-30035-2 (PMC6079037; doi:10.1038/s41598-018-30035-2)
Supplement: Supplementary file 1 — Supplementary Information [file 41598_2018_30035_MOESM1_ESM.pdf]

Supplementary Information

Hydra Mesoglea Proteome Identifies Thrombospondin as a Conserved Component active in Head Organizer Restriction

Mark Lommel, Jennifer Strompen, Andrew L. Hellewell, Gnana Prakash Balasubramanian, Elena D. Christofidou, Andrew R. Thomson, Aimee L. Boyle, Derek N. Woolfson, Kane Puglisi, Markus Hartl, Thomas W. Holstein, Josephine C. Adams and Suat Özbek

Supplementary Table S1. Mesoglea proteome of *Hydra magnipapillata*.

No	Hma identity/<br>GB Accession No./<br>Transcript ID	Protein Score	Protein	Domains (from CDD and InterProScan)<br>(/ indicates multiple domain designations for the same region)	Reference
1	Hma2.214226<br>ABG80452<br>comp25324_c0_seq1	6993	HCol-6	Collagen IV non-collagenous domain, Collagen triple-helix, C-type lectin, WAP-type 'four-disulfide core', vWFA	J. Biol. Chem. 282:6792-6802(2007)
2	Hma2.217448<br>XP_012558958.1<br>comp27401_c0_seq1	5266	Laminin, alpha	Laminin N-terminal domain, Laminin EGF, Laminin IV, Concanavalin A-like lectin/Laminin G	
3	Hma2.229572<br>XP_002168125.2<br>comp25312_c0_seq1	2971	Laminin, beta	Laminin N-terminal domain, Laminin EGF, Laminin IV type B	Dev Biol. 1994 Jul;164(1):312-24.
4	Hma2.222865<br>AAM77398<br>comp28519_c0_seq1	2332	HCol-I	Collagen triple-helix, Fibrillar collagen C-terminal domain	J. Biol. Chem. 282:6792-6802(2007)
5	Hma2.216083<br>ABG80451<br>comp18600_c0_seq1	2132	HCol-5	Collagen triple-helix, Fibrillar collagen C-terminal domain	J. Biol. Chem. 282:6792-6802(2007)
6	Hma2.232437<br>ABG80450<br>comp24511_c0_seq2	1683	HCol-3	Collagen triple-helix, Fibrillar collagen C-terminal domain, WAP-type 'four-disulfide core', vWFA	J. Biol. Chem. 282:6792-6802(2007)
7	Hma2.218921<br>NP_001296598<br>comp21769_c0_seq1	1438	Collagen type I, alpha chain-1	Collagen triple-helix, Fibrillar collagen C-terminal domain, WAP-type 'four-disulfide core'	
8	Hma2.221191<br>XP_0012557210<br>comp26030_c0_seq1	1081	Laminin, gamma	Laminin N-terminal domain, Laminin EGF, Laminin IV	

**9**	Hma2.202536<br>XP_012565750<br>comp20380_c0_seq1	882	Collagen type IV	Collagen IV non-collagenous domain, C-type lectin	J Biol Chem. 2000 Dec 15;275(50):39589-99
**10**	Hma2.213656<br>XP_012560707.1<br>comp29438_c0_seq1	467	Collagen type VI-like	vWFA	
**11**	Hma2.218942<br>XP_012558303.1<br>comp24178_c0_seq1	422	HmTSP	Concanavalin A-like lectin domain/Laminin G domain, EGF-like, TSP type-3 repeat, TSP C-terminal domain	
**12**	Hma2.208898<br>XP_002154462	171	Actin, non muscle	Actin	Gene. 1989 Dec 7;84(1):55-64
**13**	Hma2.206846<br>XP_002165741<br>comp7940_c0_seq1	125	Selenoprotein P-like	Selenoprotein P N-terminal domain, Thioredoxin-like fold	
**14**	Hma2.200137<br>XP_012560867.1<br>comp16204_c0_seq1	117	Novel	WSC superfamily	
**15**	Hma2.205448<br>XP_012564433.1<br>comp1273_c0_seq1	93	SecA-like	Protein translocase subunit SecA, SecA motor DEAD/P-loop containing nucleoside triphosphate hydrolase, Helicase C-terminal domain, Tetratricopeptide repeat	
**16**	Hma2.226267<br>NP_001274304<br>comp11507_c0_seq1	90	Nematocyst outer wall antigen (NOWA)	CAP domain, C-type lectin, Minicollagen cysteine-rich	J Cell Sci. 2002 Oct 15;115(Pt 20):3923-34
**17**	Hma2.216559<br>XP_002169906.2<br>comp18662_c0_seq1	81	Cnidarian proline-rich protein-1 (CPP-1)	Minicollagen cysteine-rich domain	J Biol Chem. 2012 Mar 23;287(13):9672-81
**18**	Hma2.203602<br>XP_004212336.2<br>comp27961_c0_seq7	75	E3 ubiquitin-protein ligase RNF213	RNF213 protein, P-loop containing nucleoside triphosphate hydrolase	
**19**	Hma2.227385<br>XP_012554267<br>comp17112_c0_seq1	74	Hemicentin-related	Immunoglobulin (Ig), TLDc	
**20**	Hma2.220431<br>XP_012557586.1<br>comp26730_c0_seq3	73	Centromere protein F-like	Coiled-coil	
**21**	Hma2.213111<br>XP_012560975.1<br>comp28013_c0_seq1	73	Kazal-type serine proteinase inhibitor	Thyroglobulin type I repeats, Kazal	
**22**	Hma2.222286<br>XP_012556696.1<br>comp28009_c0_seq5	61	Uncharacterized zoneadhesin-like protein	Growth factor receptor cysteine-rich, Chitin binding, TM helix	

**23**	Hma2.221223<br>XP_012557188.1<br>comp27373_c0_seq1	58	Centromere protein F-like	Coiled-coil
**24**	Hma2.227701<br>XP_012554110.1<br>comp27109_c0_seq1	57	Novel	Coiled-coil
**25**	Hma2.200623<br>XP_012556036.1	54	Novel	DUF4116
**26**	Hma2.223488<br>XP_002166597.1<br>comp28907_c0_seq1	52	Novel, cnidarian-restricted	
**27**	Hma2.209361<br>XP_012562660.1<br>comp28021_c0_seq1	51	Golgin subfamily B member 1-like	Coiled-coil
**28**	Hma2.225007<br>XP_012555480.1<br>comp20469_c0_seq1	51	NHL repeat-containing protein 2-like	Thioredoxin-like, TolB-like/NHL repeat
**29**	Hma2.205944<br>XP_002170701<br>comp712702_c0_seq1	50	Novel	Zinc finger C2CH-type domain, harbinger transposase-derived Nuclease domain/THAP domain
**30**	Hma2.213602<br>XP_012563309.1<br>comp24822_c0_seq3	42	Dystrophin	Spectrin repeats, PLAT/LH2, WW, EF hand, EF hand type 2/Zinc finger ZZ type
**31**	Hma2.232620<br>XP_012555788.1<br>comp25533_c0_seq4	42	Collagen type IV, alpha chain-2	Collagen triple-helix
**32**	Hma2.229657<br>XP_012566950.1	41	Novel	TM helix
**33**	Hma2.216831<br>XP_012559234.1	41	E3 ubiquitin-protein ligase RNF213-like	
**34**	Hma2.229673<br>XP_012555349.1<br>comp12798_c0_seq2	41	Novel	Alpha-L-arabinofuranosidase B/ fascin superfamily β-trefoil
**35**	Hma2.208777<br>XP_012563382.1	41	Fibrillin 1-like (partial)	
**36**	Hma2.231712<br>XP_012565417.1	40	Voltage-dependent calcium channel type A, subunit alpha-1	Ion transport domain, Voltage-dependent channel, four-helix bundle domain
**37**	Hma2.224063<br>XP_012555935.1	40	Sec1-like protein	Sec1-like, sec1-like domain 2

Unique protein hits resulting from the Orbitrap mass spectrometry analysis are listed in descending order according to their MASCOT protein score. References are given for proteins previously characterized at the protein or mRNA level. Domain annotations were performed as described in the Methods. Transcript IDs were obtained by BLAST searches of the hydra head regeneration transcriptome assemblies on https://research.nhgri.nih.gov/hydra.

Supplementary Table S2. Sequences of siRNAs, qPCR and ChIP primers.

siRNA	nucleotide sequence (5' to 3')
siGFP	TAGATGGTGATGTTAATGGGC
Scrambled siGFP	AACUCAUCGAUUCACACCGGUUUU
siTSP1	AAGATATGGTGCCTATCAGTT
siTSP2	CAGATTACTTATAAGTTTTAA
siβ-Catenin	AAGGUUAUGAUGAUGAUGAAGUU
siWnt3	AAAGAGGCUAUAACGUUAAUAUU

Gene	qPCR Primer Sequences (5' to 3')
Hm β-Catenin fw	CCTGCTATTGTTGAGGCTGGA
Hm β-Catenin rv	TCTGCAAGAGTCCTTCTAAACCA
Hm Wnt3 fw	ATTACAACAGCCAGCAGAGAAAG
Hm Wnt3 rv	TTATCGCAACGACAGTGGAC
HmTSP fw	ACCAAATGATCAAGCTGGATCCT
HmTSP rev	ACCACCAAACCTTTCAACTCCA
γ-Tubulin subunit fw	CTGCTTGTGTAGCATACTTTGAAAT
γ -Tubulin subunit rv	TCATGTTCAGCTACAAGAAATTCAC

TCF binding sites	ChIP PCR primer sequences (5' to 3')
2 juxtaposed distal sites fw	CCCGACTGTTGAAACTGC
2 juxtaposed distal sites rev	TGTTCTCAAGGCTCACGC
Proximal site fw	TTGAAGGCATTTAACAACTTGC
Proximal site rev	TGCCCAAATGTAAAGTTCTGTG
Intronic site fw	GACAAAACCAAAGAAAAAATG
Intronic site rev	TCCCTTGTAATCAGGTTAG
Control fw	ACTTTCATATAAGGCCGCTTC
Control rev	CCCAGCGATATAGAGACTGC

Supplementary Fig. S1

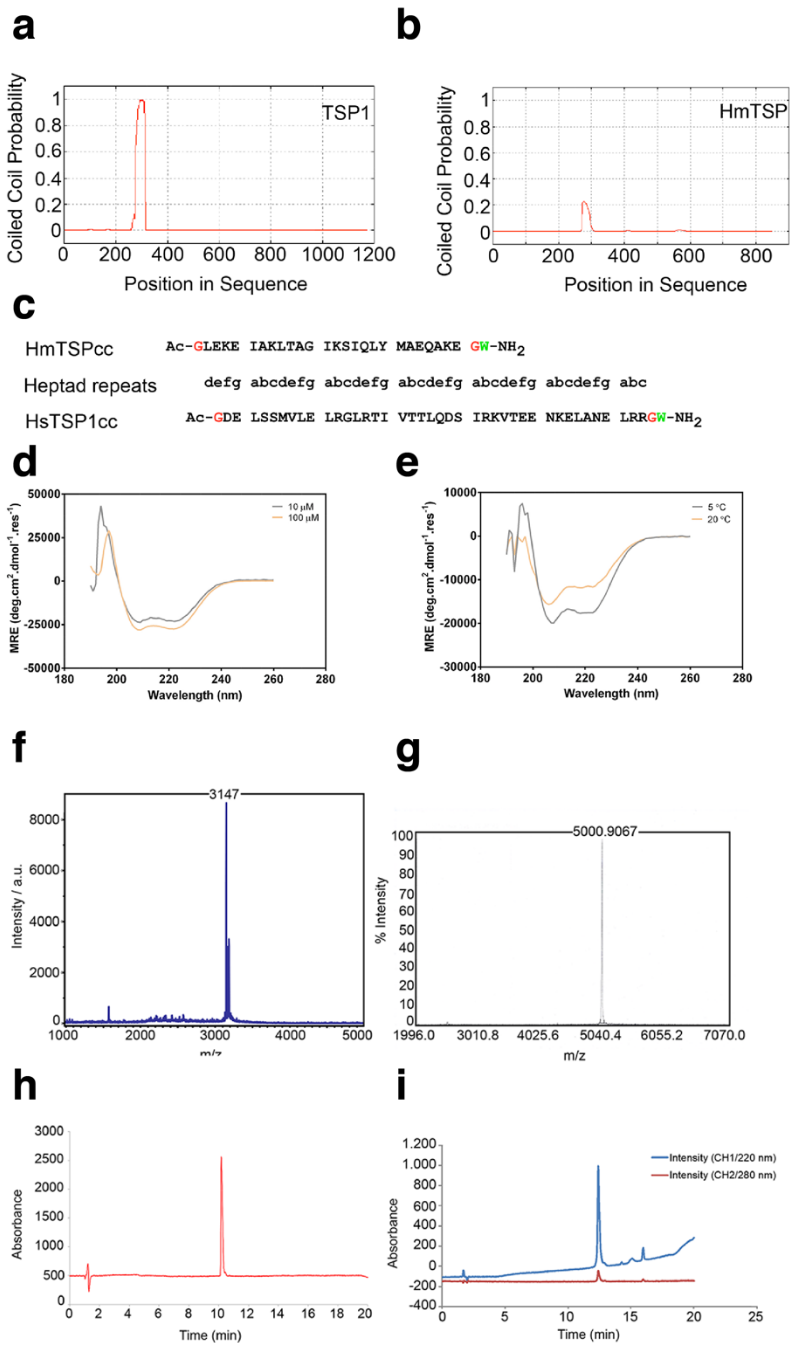

Fig. S1. Preparation of synthetic peptides from the oligomerization regions of TSP1 and *Hydra* TSP. a, b. Coiled-coil region probabilities in human TSP1 (a) or *Hydra* TSP (b), as predicted in MARCOIL. **c**. Protein sequences of the synthetic peptides corresponding to the predicted oligomerization regions of *Hydra* TSP (HmTSPcc) and human TSP1 (HsTSP1cc). In each peptide, the native sequence is given in black, added glycines in red and added tryptophan in green. The heptad repeat phase is predicted from MARCOIL. **d, e**. Circular dichroism spectra for HsTSP1cc (d) or HmTSPcc (e) peptides. HsTSP1cc was analyzed at two concentrations at 5 °C. HmTSPcc was analyzed at 100 μM at 5 °C and 20 °C. MRE = mean residue ellipticity. **f, i**. Quality control for the synthetic peptides**.** Spectra from MALDI-TOF mass spectrometry for HmTSPcc (f) and HsTSP1cc (g) peptides. Each identifies a single peak consistent with the predicted mass for each peptide. **h, i.** Traces from RP-HPLC of HmTSPcc (h) and HsTSP1cc (i) peptides, demonstrating the purity of the preparations.

Supplementary Fig. S2

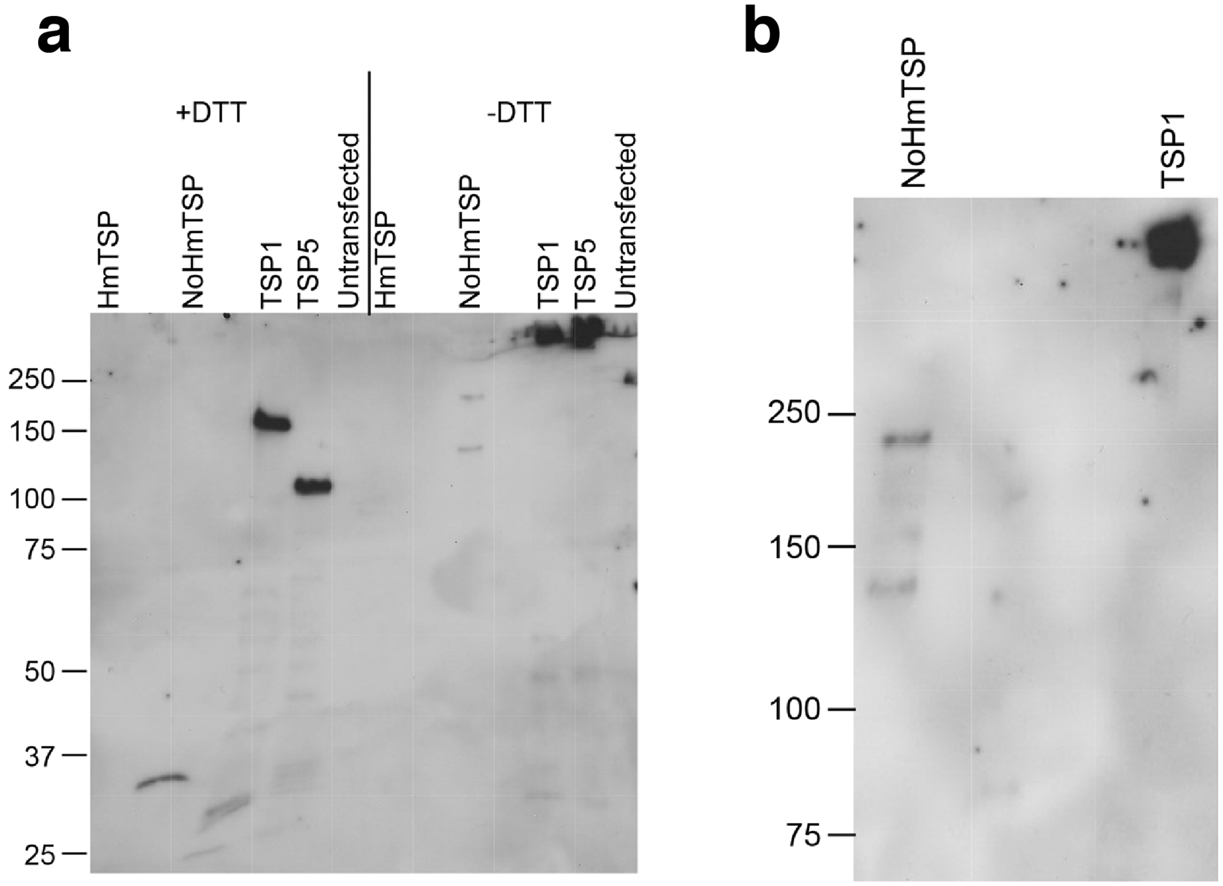

Fig. S2. Uncropped Western blot images used in Fig. 3d. **a.** Recombinant NoHmTSP.V5 and human TSP1.V5 and TSP5.V5 analyzed under reducing and non-reducing conditions, including lanes presented in Fig. 3D, left panel. **b.** Recombinant NoHmTSP and human TSP1 analyzed under non-reducing conditions as presented in Fig. 3d, right panel. Each immunoblot was probed with V5 antibody. To improve resolution of high-molecular weight proteins, some molecular weight markers were run off the bottom of each gel a and b as indicated.

Supplementary Fig. S3

a

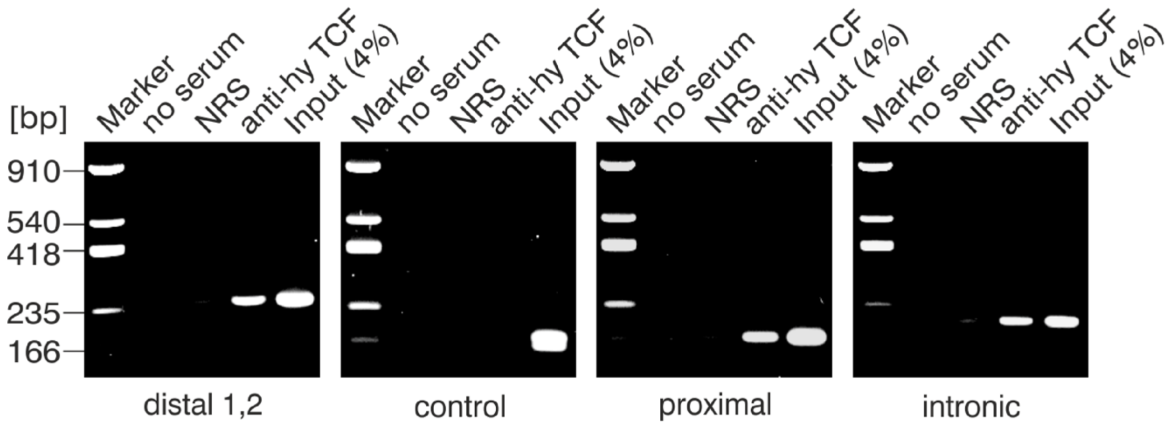

b

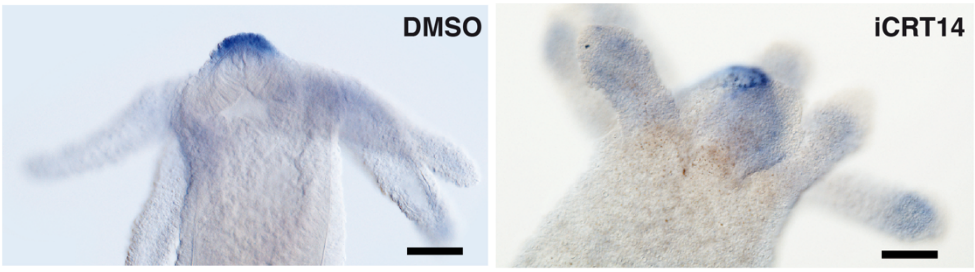

Fig. S3. Regulation of *HmTSP* expression by β-Catenin. a. Replication of the ChIP experiment analyzing the *Hydra TSP* promoter region (compare Fig. 7b) using an independent chromatin preparation from whole *Hydra* animals. A polyclonal antibody directed against *Hydra* TCF was used for precipitation, followed by PCR amplification of the indicated fragments from the *TSP* regulatory region including a region without TCF binding site (control). Reactions with normal rabbit serum (NRS) or total chromatin (Input) were used as further controls. PCR products were resolved by agarose gel electrophoresis and visualized by ethidium bromide staining. **b.** Representative *HmTSP* expression in DMSO-treated control animal at 72hrs (left) or animal treated with 50 µM of β-Catenin inhibitor iCRT14 for 24 h and fixed after 72 hrs. Bars: 500 µm.
